# Unraveling the mystery of stuttering: clinical and physiological insights into its manifestation

**DOI:** 10.3389/fnhum.2026.1700499

**Published:** 2026-04-08

**Authors:** Jalal Majid Jalil, Asmaa Abbas Ajwad, Diyar Majid Jalil

**Affiliations:** 1 College of Medicine, University of Diyala, Baqubah, Diyala, Iraq; 2 College of Pharmacy, Al-Yarmouk University, Baqubah, Diyala, Iraq

**Keywords:** anticipation, Auditory-speech motor control, developmental differences, dopamine dysregulation, right inferior frontal gyrus, self-monitoring system, situational variability

## Abstract

Stuttering is a complex neurodevelopmental speech disorder characterized by involuntary sound and syllable repetitions, prolongations, and speech blocks, accompanied by marked variability across linguistic, emotional, and situational contexts. Although numerous hypotheses have been proposed to explain its underlying mechanisms, many have encountered a fundamental limitation: the difficulty of coherently accounting for the full range of clinical, developmental, and neurobiological features observed in people who stutter. In response to this gap, the present work proposes a comprehensive, integrative hypothesis that seeks to unify the diverse physiological and clinical manifestations of stuttering within a single neurobiological framework. This model aims to link moment-to-moment fluctuations in speech behavior with neurodevelopmental alterations, offering a plausible mechanistic account for a wide spectrum of core phenomena. These include the pronounced situational variability of stuttering severity; the developmental shifts from repetitions to blocks; the transition of disfluencies from function words to content words; the tendency for stuttering to occur on key words in a sentence; and the consistently lower rates of spontaneous recovery observed in males compared to females. Furthermore, the proposed framework seeks to explore potential common mechanisms underlying the widespread structural, metabolic, and functional brain changes documented in stuttering, while considering whether these abnormalities may reflect primary contributors or secondary, compensatory adaptations. In particular, the model seeks to address a long-standing debate regarding the role of the right inferior frontal gyrus, examining whether its engagement is more consistent with a causal contribution to speech disruption or with an adaptive response to impaired speech–motor control. By integrating neurodevelopmental, physiological, and clinical evidence, this hypothesis offers a unifying perspective on key features of stuttering while proposing a neurobiological model whose assumptions and hypotheses can be empirically tested and evaluated in future experimental studies.

## 1 Introduction

Although often defined in terms of speech disfluency, developmental stuttering carries consequences that reach far beyond the act of speaking, exerting a broad and often enduring psychosocial impact that can begin early in life. During childhood, when peer acceptance and social comparison play a central role in shaping self-esteem, children who stutter (CWS) are more likely to experience social rejection, reduced peer status, and increased exposure to bullying, and are less frequently perceived as popular or as leaders within their peer groups (Davis et al., 2002; Berchiatti et al., 2021). Such early social disadvantages coincide with elevated internalizing vulnerability, with school-aged CWS showing markedly increased risk for anxiety disorders, including “six-fold increased odds” of social anxiety disorder relative to their fluent peers (Iverach and Rapee, 2014; Iverach et al., 2016).

As these experiences accumulate throughout development, their impact often becomes more closely tied to self-evaluation and identity formation. For many individuals, these patterns persist into adulthood, manifesting as chronic feelings of shame, avoidance of social interaction, and restrictions in participation across educational, interpersonal, and occupational domains (Türkili et al., 2022; Alatawi and Good, 2025). Evidence from adolescence also indicates that greater stuttering severity is associated with lower domain-specific and global self-esteem, alongside heightened sensitivity to peer evaluation and emerging self-stigma (Butler, 2013; Adriaensens et al., 2015).

The functional consequences of these psychosocial challenges are particularly evident in employment contexts. Survey data indicate that more than 70% of adults who stutter (AWS) believe their speech difficulties reduce their chances of being hired or promoted (Klein and Hood, 2004), while population-level analyses demonstrate measurable labor market disadvantages, including an earnings deficit exceeding $7,000 for AWS and increased underemployment among women who stutter (Gerlach et al., 2018). At the same time, greater stuttering burden has been linked to poorer mental health outcomes, with higher levels of depression, anxiety, and stress reported among adults experiencing greater adverse impacts (Engelen et al., 2024). Converging evidence further underscores the clinical significance of these associations, as elevated depressive symptoms and an increased risk for suicidal ideation have been documented in some subgroups of people who stutter (PWS) (Briley et al., 2021; Tichenor et al., 2023).

Despite its well-documented psychological consequences, stuttering remains a puzzling neurodevelopmental disorder of unclear etiology, characterized by substantial heterogeneity in its clinical presentation. In addition to classical developmental stuttering, several clinically distinct forms have been described, including neurogenic, psychogenic, and pharmacologically induced stuttering. Although there is overlap in both symptoms and neurological causes across these forms (Theys et al., 2024), clear and systematic differences also exist among them. Non-developmental forms are typically preceded by identifiable precipitating events, such as neurological injury, psychological trauma, or medication exposure. Moreover, neurogenic stuttering tends to show greater consistency across speaking tasks and communicative contexts, whereas psychogenic stuttering may exhibit marked improvement following psychological intervention (Cruz et al., 2018; Zunic et al., 2021). For reasons of conceptual precision and interpretive clarity, the present work focuses specifically on classical developmental stuttering.

Classical developmental stuttering is conventionally defined by its overt speech disruptions, including repetitions, prolongations, and speech blocks. While these features constitute the most recognizable clinical manifestations of the disorder, limiting the definition to speech-level phenomena alone overlooks a second, equally fundamental characteristic: situational variability. Stuttering severity is known to fluctuate substantially across speaking contexts, communicative demands, and time, a phenomenon that has been consistently documented over decades of research (Bloodstein, 1949; Shulman, 1955; Yaruss, 1997; Constantino et al., 2016; Tichenor and Yaruss, 2021; Usler, 2022; Lei et al., 2024; Rasoli Jokar et al., 2025). This marked variability has posed a persistent challenge to theoretical models of stuttering and has motivated the use of multiple speech samples across diverse contexts to obtain ecologically valid assessments of fluency (Tichenor and Yaruss, 2021). Given its central relevance, situational variability warrants explicit consideration as a core feature of developmental stuttering. The present work therefore begins by examining this phenomenon in depth, as we contend that it holds a crucial key to understanding both the emergence of stuttering and the manner in which it manifests across different speaking situations.

## 2 Situational variability in stuttering

### 2.1 Definition and types

Although a substantial body of research has examined situational variability, many studies have relied on closely related definitions, often describing the phenomenon through brief explanations rather than a formalized definition. To enhance conceptual precision, we therefore propose a unified definition of situational variability grounded in the cumulative evidence and historical literature mentioned above. Accordingly, situational variability can be defined as any noticeable change in stuttering frequency and/or severity that occurs when an individual is exposed to different speaking situations, contexts, or tasks, as well as across time, including day-to-day variation.

Situational variability should not be regarded as a random or unsystematic fluctuation in stuttering frequency/severity. Rather, the extensive body of research examining this phenomenon has progressively clarified its structure and components, allowing for meaningful classification of its patterns. Based on the collective findings of this literature, situational variability can be broadly divided into three categories.

The first of these is what we call *stereotypical variability*. Stereotypical variability refers to speaking situations in which a consistent and predictable pattern of stuttering behavior has been repeatedly documented. In these situations, both clinicians and PWS can reasonably anticipate whether stuttering will markedly decrease or significantly worsen in frequency and/or severity. This category can be further subdivided into two primary types.

The first type comprises fluency-inducing conditions, which are consistently associated with a substantial reduction in stuttering severity/frequency. These include self-talk/talking when no one is present (Bloodstein, 1949; Langová and Šváb, 1973; Jackson et al., 2021), singing (Wan et al., 2010), choral reading (Freeman and Armson, 1998; Dechamma and Maruthy, 2018; Meekings et al., 2023), and entering states of euphoria or intense focus/engagement, adopting novel or altered speech patterns, or brief episodes of emotional outburst (Usler, 2022). In such conditions, stuttering is reliably attenuated, often to a marked degree.

The second type includes conditions that exacerbate stuttering, which are well known to intensify stuttering severity/frequency. These typically involve heightened communicative demand or social evaluative pressure, such as speaking in front of an audience (Porter, 1939; Hahn, 1940; Moïse-Richard et al., 2021), addressing an authority figure (Sheehan et al., 1967), introducing oneself, including saying one’s own name (Usler, 2022).

Across both fluency-inducing and stuttering-exacerbating situations, a robust and directional change in stuttering severity is observed: improvement in the former and deterioration in the latter. Importantly, this effect appears to be broadly shared among PWS, indicating a common underlying sensitivity to these situational conditions. However, within this shared directional response, notable variability remains. For example, although nearly all participants in studies examining speaking alone demonstrate noticeable improvement in fluency, the magnitude of this improvement differs among individuals. Some reach near-complete or complete fluency, whereas others continue to stutter, albeit with reduced severity (Bloodstein, 1949; Langová and Šváb, 1973; Jackson et al., 2021). A similar pattern has been reported in choral reading; an improvement was observed in all participants; however, the magnitude of stuttering reduction varied significantly among individuals, ranging from approximately 77.6 to 99.7% (Freeman and Armson, 1998) and 90 to 100% (Dechamma and Maruthy, 2018; Meekings et al., 2023). During singing, all participants exhibited a strong reduction in stuttering, although minor inter-individual differences in improvement persisted, estimated at around 10% (Wan et al., 2010).

The second category, which we refer to as *time* var*iability*, describes noticeable changes in stuttering frequency/severity across different temporal scales. Such variability may occur rapidly within a single day or more gradually across longer periods, including days, weeks, or even months (Yaruss, 1997; Constantino et al., 2016; Tichenor and Yaruss, 2021; Lei et al., 2024).

The third and final category in our classification is *individual variability*, which refers to the observation that different PWS may respond differently to the same speaking situation or task in terms of changes in stuttering frequency and/or severity. This category captures inter-individual differences in how situational demands influence stuttering behavior and can be further divided into three subtypes.

The first subtype involves a broadly similar directional response across individuals, with variability emerging primarily in magnitude rather than direction. A clear example of this pattern is observed in responses to fluency-inducing conditions, where most PWS demonstrate a marked degree of improvement, yet the extent and strength of this improvement may vary between individuals.

The second subtype reflects genuinely divergent responses to the same situation, whereby identical contexts or tasks elicit opposite effects across different individuals, excluding situations that are already well established as universally fluency-inducing or stuttering-exacerbating. As described by Rasoli Jokar et al. (2025), activities such as traveling or playing with peers were reported to increase stuttering severity in some children, while in others, these same activities were reported to reduce apparent stuttering severity. Similar inter-individual contrasts have also been noted in earlier work, including observations reported by Yaruss (1997).

The third subtype concerns variability in the qualitative characteristics of stuttering itself, both within and between individuals. Specifically, the particular sounds, syllables, or words on which stuttering occurs may differ among PWS. Moreover, within the same individual, these loci of stuttering are not fixed and may shift over time, such that previously difficult sounds or words become less problematic while new ones emerge (Tichenor and Yaruss, 2021).

Interestingly, despite the substantial body of evidence and the critical importance of this feature, situational variability is rarely incorporated into formal definitions of stuttering in the majority of the literature. Yet this very feature has served as the foundation upon which numerous hypotheses have been constructed and, notably, the same foundation upon which many of them have been challenged or rejected. Accordingly, to unravel the mystery of stuttering, the present work deliberately begins with its most elusive feature, revisiting a long-standing question that has accompanied the field for decades: Why does stuttering appear to be situationally variable?

### 2.2 The historic question that sparked it all

When examining the earliest studies and hypotheses about stuttering, it is striking that many of them also began from this very phenomenon. In the work of Fletcher (1914), stuttering was shown to vary markedly with who is listening and the speaking setting (often easier in private than under scrutiny); Tompkins (1916) interpreted these shifts as fear-driven, misdirected conscious effort interfering with otherwise automatic speech and noted strong improvements in singing and unison speech; and Swift (1915) suggested a mechanistic clue in atypical conscious imagery during speaking. Taken together, these early accounts were among the first to draw attention to what would later be termed the situational variability of stuttering.

In recent years, several well-known hypotheses have been advanced to explain stuttering. These include the brain energy hypothesis (Alm, 2021), the speech rhythm hypothesis (Etchell et al., 2014), the striatal dysfunction hypothesis (Maguire et al., 2020), and the overreliance-on-auditory-feedback hypothesis (Civier et al., 2010). Each of these frameworks has contributed meaningful progress and has strengthened our understanding of stuttering by illuminating specific mechanisms that may be involved.

At the same time, none of these accounts appears to have sufficient scope to explain the full phenotype of stuttering, including situational variability across the wide range of speaking contexts discussed above. In most models, a remaining gap is almost inevitable; it may reflect contradictions in accounts of how stuttering emerges across speaking situations, an incomplete integration of established neurophysiological and neuroanatomical alterations, or a framework whose explanatory aim is restricted to a single component rather than the full clinical phenotype.

From our perspective, any effort to identify the cause of stuttering should, at least in principle, account for all major stuttering phenomena and symptoms before being treated as an etiological explanation and tested empirically. In this regard, and because stuttering has a precise neurological signature, it is essential to first examine what is already established in adults who stutter (AWS) regarding brain structure and overall patterns of neural activity.

### 2.3 Brain changes in adults and CWS

Neuroimaging studies consistently show that AWS exhibit reliable structural and functional brain differences. Functionally, reduced activation is often observed in left hemisphere language and speech–motor regions, alongside atypical basal ganglia involvement (Maguire et al., 2020). Although activity in several cortical regions may normalize under fluency-enhancing conditions such as choral reading, striatal activity can remain abnormally low, which has been interpreted as compatible with impaired feedforward control within left hemisphere speech networks (Chang et al., 2019). Structurally, gray matter reductions have also been reported in the striatum, specifically the left caudate nucleus (Sowman et al., 2017).

In parallel, neuroimaging studies have reported increased recruitment of right-lateralized control and salience systems during speech in AWS, most consistently involving the right inferior frontal gyrus, anterior cingulate cortex, right dorsolateral prefrontal cortex, and right anterior insula, with additional involvement of limbic regions such as the amygdala in some reports (Chang et al., 2009; Kaganovich et al., 2010; Budde et al., 2014; Belyk et al., 2015; Neef et al., 2018; Toyomura et al., 2018; Jackson et al., 2022). Consistent with atypical sensory monitoring, AWS also show diminished pre-speech auditory suppression (Daliri and Max, 2018), and the left superior temporal gyrus often exhibits reduced activation and abnormal connectivity during natural speech, with partial normalization under fluency-enhancing conditions (Garnett et al., 2022).

It is noteworthy that many of the neural alterations reported in AWS appear to represent a developmental continuation of patterns already detectable in childhood. In preschool-aged children with persistent stuttering (3–5 years), Chow et al. (2023) reported reduced gray matter volume in the striatum, specifically the putamen and nucleus accumbens, slower development of the left inferior frontal gyrus, and reduced white matter volume in major tracts including the bilateral corona radiata, superior longitudinal fasciculus, and corpus callosum. These findings are consistent with broader evidence implicating the basal ganglia–thalamocortical (BGTC) loop in stuttering across studies (Sommer et al., 2002; Guenther, 2006; Kell et al., 2024; Beal et al., 2013; Foundas et al., 2013; Connally et al., 2014; Civier et al., 2015; Chang et al., 2008, 2015, 2019).

A similar developmental continuity may also apply to right hemisphere involvement; Neef et al. (2023) reported that the right posterior inferior frontal cortex (pars opercularis) in CWS aged 3–11 shows a mixed connectivity profile, with enhanced coupling to insula and somatomotor regions implicated in motor control and inhibition, alongside weaker coupling with components of the dorsal attention network, which supports attentional and top–down cognitive regulation.

### 2.4 Contemporary hypotheses and their limits

Taken together, these investigations reveal a clearly delineated disturbance predominantly affecting the left hemisphere, particularly regions involved in speech production. However, while many of these cortical areas demonstrate context-dependent variability in activation across speaking situations, one structure stands out as unusually consistent: the striatum. Structural and functional abnormalities within the striatum have been documented as early as 3–5 years of age and appear to persist into adulthood. This continuity initially led to the assumption that stuttering arises primarily from a deficit within the left hemisphere speech production network.

Yet, such a fixed deficit alone is clearly insufficient. If stuttering were solely the consequence of a stable impairment in speech–motor regions, the pronounced phenomenon of situational variability would not be observed. The marked fluctuations in fluency across contexts indicate that stuttering cannot be reduced to a static dysfunction, even though abnormalities in left hemisphere speech regions are well established. The disorder is therefore more complex than a simple impairment of speech production mechanisms.

This realization prompted the exploration of alternative explanations. One early line of reasoning focused on speech rhythm (Etchell et al., 2014), motivated by the observation that stuttering often diminishes dramatically or even disappears in conditions such as singing and choral reading. These findings suggested the presence of a disrupted internal timing or rhythmic mechanism, with external rhythmic cues compensating for this deficit and thereby improving fluency. However, this hypothesis left critical questions unresolved. How, for example, can it explain the reduction of stuttering during self-speech or during moments of euphoria and intense emotional arousal?

More recently, Meekings et al. (2023) proposed findings indicating that during choral speech, PWS had an increased speech rhythm frequency, whereas neurotypical speakers had a decreased frequency. This opposite pattern challenges the hypothesis that PWS achieve fluency by matching their partner’s rhythm. Instead, fluency may result from the temporary suspension of compensatory strategies rather than aligning with external rhythms. Thus, fluency may not be directly dependent on rhythm imitation.

In an attempt to explain why stuttering decreases during self-speech and other low-pressure situations, the brain energy hypothesis was introduced (Alm, 2021). According to this view, a generalized reduction in neural energy production limits the brain’s ability to support speech under cognitively demanding or stressful conditions, whereas simpler, low-pressure contexts remain manageable (e.g., self-talk). This hypothesis offered an appealing explanation for several aspects of situational variability. However, much like the rhythm hypothesis, it encountered significant limitations. It failed to account for the absence of stuttering during singing and choral reading, as well as during euphoric or highly emotional states, contexts in which neural energy consumption would presumably be elevated. Moreover, it could not explain individual variability, whereby different individuals exhibit opposite fluency responses to the same speaking situation.

Attention then shifted to the overreliance-on-auditory-feedback hypothesis (Civier et al., 2010). This model proposes that individuals who stutter rely excessively on auditory feedback during speech, rather than on efficient feedforward motor planning. Because auditory feedback is inherently slower than motor execution, this overdependence renders speech vulnerable to delays, hesitations, and breakdowns. While this framework successfully explains several fluency-enhancing conditions, it again falls short in critical areas. It does not adequately account for individual variability, nor does it explain the emergence of speech blocks. Furthermore, it localizes the core deficit almost exclusively to left hemisphere speech and auditory regions, while neglecting the right hemisphere overactivity.

This raises a critical question: is it possible to formulate a hypothesis that integrates these neural abnormalities while also offering a coherent and mechanistically plausible account of situational variability and its associated features?

## 3 From neural abnormalities to situational variability: a unified hypothesis

### 3.1 The consistent theme across all experiences

We begin with the aspect of stuttering that is supported by the strongest and most consistent empirical evidence: atypical developmental changes in the left hemisphere, particularly within speech production regions and their adjacent cortical networks.

Based on the information we have discussed regarding the evident abnormalities in the left hemisphere, particularly in the BGTC and the striatum, along with auditory regions like the LSTG, these abnormalities, regardless of their specific nature, lead to what we refer to as error signals. Error signals refer to any issues or disruptions that occur between the functioning and communication of auditory–speech–motor systems.

Despite these abnormalities, as is evident, they seem insufficient to maintain stuttering consistently. A prominent example of this is the complete disappearance or significant reduction in stuttering when speaking to oneself or in solitude. Hence, the emergence of stuttering in situations such as public speaking, delivering an important message, or speaking to someone in authority—common scenarios for stuttering—suggests that the disruption in these regions is fundamentally weak, below the threshold required for stuttering to emerge (Brocklehurst et al., 2013).

Therefore, for stuttering to manifest, some other component must intervene. Here, we are presented with two possibilities: either this second mechanism exceeds the threshold, triggering stuttering, or it supports a different mechanism that causes stuttering without necessarily surpassing a threshold in speech production regions.

To investigate this mechanism, and based on our consideration of all types of issues that may arise along the auditory–speech–motor pathway as error signals, the first question that comes to mind is how does the brain handle these error signals? How does it respond to them?

### 3.2 The self-monitoring system as the brain’s error-handling mechanism

The brain seems to possess a specialized mechanism for detecting and correcting error signals, referred to as the self-monitoring system (SMS). The SMS, as outlined by (Nozari, 2025), Nozari et al. (2011) and Arenas (2017), a collection of cognitive and neural processes that continuously assess and regulate speech production. It identifies discrepancies between expected and actual speech outcomes by employing mechanisms such as conflict monitoring and forward models (predicting sensory feedback from speech actions). This system is crucial for ensuring fluent speech, adjusting speech plans in response to linguistic conflicts, motor planning issues, and the influence of emotional and social factors. It integrates both internal cognitive feedback and external feedback to optimize the accuracy of speech production.

The SMS is a continuous, automatic process that operates persistently throughout speech production. It does not activate or deactivate at specific moments but functions continuously as a natural mechanism for detecting and correcting errors. It should be viewed as an inherent, supportive system in the process of speech production, particularly within the framework of this discussion (Nozari et al., 2011).

This system is characterized by its distinctive ability to function primarily subconsciously, continuously tracking speech production and making automatic adjustments without conscious involvement. However, when significant errors or discrepancies are detected, the system can be upregulated to conscious awareness, allowing for intentional attention and correction of the speech output (Nozari, 2025).

Up to this point, we have been dealing with a natural and supportive framework for speech production, where speech normally proceeds under largely subconscious control. Within this framework, conscious attention to speech can be understood as an additional strategy employed by the system to further support speech production by recruiting perceptual regions and allocating explicit attentional resources to speech.

However, the salient feature emerging from the shift from subconscious to conscious processing raises a critical concern. This feature appears to be consistently present in most situations associated with an increase in stuttering and notably absent in situations where stuttering is markedly reduced or disappears.

For instance, in singing, attention is redirected toward music, melody, and the reformulation of speech within a new rhythmic and prosodic structure. In choral reading, attention is anchored to rhythm and temporal alignment with others’ speech. In states of euphoria or deep engagement, attentional resources are almost entirely captured by the external stimulus. Similarly, during intense emotional arousal, the system’s attentional capacity is strongly oriented toward the external emotional trigger.

Across all these conditions, we observe a common pattern: the conscious component of the SMS is either reassigned or “hijacked” away from speech itself, allowing error detection and correction to proceed subconsciously. Speech therefore remains fluent. In contrast, the system can be upregulated into conscious awareness in virtually all situations in which stuttering emerges: speaking in front of others, addressing authority figures, delivering an important message, self-presentation, or even stating one’s own name.

At first glance, this suggests that the transition from subconscious error adjustment to conscious error adjustment constitutes the core mechanism underlying the emergence of stuttering. Yet, this explanation alone is insufficient, as it fails to account for one of the most robust fluency-enhancing conditions: speaking to oneself or speaking alone. In this context, an individual may direct conscious attention to speech and still speak fluently.

This observation necessitates the introduction of a second critical component, which, together with conscious attention, appears to form the core mechanism governing the appearance and disappearance of stuttering. This component is social evaluation: the process by which individuals judge themselves based on perceived social standards, expectations, and feedback from others, often influencing emotions, behavior, and self-concept. Social evaluation is present in all situations where stuttering emerges and, crucially, requires the presence of one or more listeners. It is markedly reduced during choral reading, where the individual voice is masked by others. Therefore, no individual or special attention is directed toward any single person, as they are considered part of the group. It also diminishes significantly during states of euphoria, emotional intensity, or deep engagement, where awareness of the self and even the self as an entity fades. In singing, self-evaluation may still be present, yet conscious attention to error signals is largely absent, as attentional resources are fully allocated to melody and rhythm.

Thus, what appears to explain the stereotypical variability category of stuttering is the interaction between two factors: conscious error monitoring and social evaluation. Wherever these two factors co-occur, stuttering emerges. Wherever one or both are absent, stuttering is significantly reduced and may disappear entirely in certain individuals or contexts. The critical question, therefore, is what these two factors induce at the neural level? What is happening within the brain when they co-occur?

### 3.3 Neural-level interpretation

The error signals originating within speech production regions are initially detected by the SMS. From this point, two distinct processing routes can be identified. In route 1, in the absence of one or both factors (conscious attention and social evaluation), these signals are processed by the SMS as *ordinary error signals*. They are resolved either subconsciously or consciously but without the presence of social evaluative pressure. In route 2, when both factors are simultaneously present, a qualitatively different process emerges. Although the SMS still detects the same error signals, it no longer treats them as neutral error information. Instead, the co-presence of conscious attention and social evaluation forces the system to reinterpret these signals as warning signals.

In other words, speech context transforms error signals from neutral markers of deviation into signals imbued with threat relevance. This involves a shift from subconscious to conscious control, mediated by higher-order neural regions. This process can be summarized as follows:

Error signals → no conscious error monitoring / no social evaluation → error signals processed within monitoring system.
Error signals → social evaluation + conscious error monitoring → warning signals → recruitment of additional regions.

In this framework, stuttering is not the result of defective error detection per se, but rather of a context-dependent escalation of error signals into warning signals, driven by the convergence of conscious monitoring and social evaluative processing.

The question that follows is: what constitutes these warning signals, and how can their function be understood? In our hypothesis, warning signals are not independent signals per se, but rather a reinterpretation of error signals broadcast by the SMS, indicating that these signals carry heightened contextual significance. This heightened significance emerges when the speech context is socially or personally salient, such as during social evaluation, perceived importance of the listener, performance-related expectations, fear of failure, and the desire to avoid negative attention. Within such contexts, these cognitive and affective factors imbue error signals with emotional weight, leading the SMS to reclassify them as warning signals. These warning signals operate within a defensive framework, whereby the system attempts to recruit additional neural resources and allocate increased attentional focus in the service of caution, precision, and control, with the goal of producing fluent speech and achieving the intended communicative impression or goal of the speaker.

To clarify this further, specific examples can demonstrate how they impact critical operational factors, including social evaluation and conscious error monitoring.

When PWS talk to someone they are comfortable with, they pay less attention to speech errors, and social evaluation indicates that it is acceptable to stutter, resulting in a notable reduction in stuttering. However, when PWS talk to that same person but need to deliver an important message or convey something precisely, they pay much more attention to speech errors, and social evaluation is heightened due to the pressure to speak correctly and fluently, leading to an increase in stuttering. In another example, at critical moments—such as a problem or an issue of great importance in the life of PWS, or speaking in front of a very important person—two scenarios may occur.

In the first scenario, stuttering may become extremely pronounced. The interpretation here is that attention is heavily focused on error monitoring and heightened social evaluation, which amplifies speech disruptions. In the second scenario, there may be a sudden reduction in stuttering, with speech flowing smoothly and without interruption. Our interpretation is that the speaker’s attention is fully directed toward the situation itself, while the conscious monitoring system is temporarily overridden by the external demands and context. As a result, the speaker momentarily “forgets” themselves and stops focusing on errors, engaging fully with the idea, the person, and the situation. This explains why, within the same situation, stuttering may intensify in one individual while diminishing in another. This variability largely depends on where the speaker’s attentional focus is directed; when this focus is on error monitoring and converges with social evaluation, stuttering is more likely to emerge.

### 3.4 Why threshold explanations remain insufficient

There is a perspective that will emerge here, suggesting that these warning signals may represent the very point that reflects the assumed threshold in speech production areas and triggers the manifestation of stuttering (Brocklehurst et al., 2013). This is a reasonable view; however, there is a distinctive feature that tends to weaken this argument and cast doubt on it.

A notable pattern emerging from clinical observations and empirical reports is that stuttering is more likely to occur on “critical” words—those that carry greater communicative importance within an utterance—rather than being randomly distributed across speech (Kaasin and Bjerkan, 1982). PWS can often produce alternative or less contextually appropriate words fluently, yet experience breakdowns precisely on the word they judge to be the “correct,” most meaningful, or most contextually appropriate response. For instance, a person may block on the straightforward request ‘Can you help me?’ yet produce a less direct and more circuitous formulation such as ‘Sorry… um… I have a question’ with relative ease.

This phenomenon suggests that perceived importance increases the salience of specific words within the SMS, thereby increasing the likelihood of dysfluency. It is not a coincidence that stuttering often occurs on important words—the most meaningful and relevant in the sentence—while it decreases for words of less significance in the context (Usler, 2022). This interpretation aligns with findings that PWS frequently anticipate upcoming moments of stuttering (Silverman and Williams, 1972; Jackson et al., 2018, 2020) and often engage in word substitutions or reformulations to avoid anticipated difficulty. Such avoidance is less applicable in situations where only one lexical item is appropriate, such as providing one’s name or labeling an object. In these cases, the warning signal represented by the SMS appears to be disproportionately focused on specific words rather than uniformly applied across entire sentences, transforming the target word into an anticipated “fear word.” Social evaluation pressure and conscious monitoring can amplify error signals into general warning signals. However, the involvement of cognitive, emotional, and logical processes can narrow these signals to the specific words that seem most important within a sentence. Importantly, this phenomenon does not need to occur exclusively at the moment of speaking; it can arise well in advance (Jackson et al., 2015).

For example, consider a person who stutters preparing to meet someone new in the coming days. They may begin to anticipate potential sources of embarrassment, such as the likelihood that the first question will be, “*What is your name?*” If they falter or cannot produce the answer fluently, they fear appearing socially awkward. In this scenario, higher-order cognitive and knowledge-based regions have already determined which word is most important in the sentence. Consequently, when the situation arises and the question is asked, the system is activated, generating warning signals that reflect the contextual importance, guided by the higher-order structures that previously identified the critical word. The entire attentional focus converges on that word. This mechanism can operate both in real-time during speech and in advance, as illustrated in the example above.

This idea has been discussed for decades within the stuttering literature. Bloodstein (1955) in his discussion of information-load accounts of stuttering, proposed that moments of low predictability in spoken sentences impose heightened uncertainty and communicative responsibility on the speaker. At such points, the speaker alone must supply essential information, increasing vulnerability to speech disruption. Consistent with this observation, words that PWS identify as feared or anticipated continue to elicit stuttering even months after the participants have identified them (Mersov et al., 2018).

Another important and interesting feature that has been widely discussed is that, even during moments of severe difficulty such as when asked, “*What is your name?*” PWS often remain capable of producing fluent but contextually inappropriate speech. For instance, they may delay providing their name by beginning with a carrier phrase such as *“My name is…”* and inserting a pause before producing the actual word, or they may rely on circumlocutions or offer an alternative inappropriate lexical item, such as saying another name. Another supporting example is the repetition of entire sentences or whole words. PWS may repeat a full sentence to gain access to a single target word within it. Additionally, PWS frequently adopt alternative speaking strategies to compensate for or prevent anticipated difficulty, including employing easy onset to begin speaking, using fillers or sentence starters, and interrupting the communication partner (Van Riper, 1982; Vanryckeghem et al., 2004; Arenas, 2012; van Lieshout et al., 2014; Jackson et al., 2015). This means that even during moments of speech difficulty, PWS can still produce fluent speech, and the difficulty itself appears to be focused on specific words perceived as the most appropriate or contextually relevant within the utterance.

While the VRT hypothesis by Brocklehurst et al. (2013) has made a valuable contribution to the scientific understanding of stuttering, it does not fully account for several key characteristics of the disorder. Stuttering is often word-specific, disproportionately affecting socially, emotionally, or communicatively salient words, while adjacent words remain fluent or can be substituted. PWS frequently employ dynamic compensatory strategies, such as repeating entire sentences to access a single target word, inserting carrier phrases with pauses, or reformulating sentences in real time. This observation challenges the notion that the release threshold is localized solely within speech production areas. If that were the case, speakers should not be able to substitute words easily or produce contextually inappropriate alternatives fluently, and stuttering would be expected to occur more uniformly across speech rather than selectively targeting a single word while other words remain fluent at the moment of blocking or repetition.

What we have reached so far is the conclusion that the warning signals act as a protective mechanism in the speech system, designed to help ensure smooth speech by recruiting other higher-level cognitive and attentional processes. However, within this recruited system, there seems to be a component that does not function normally. Instead of simply supporting speech, this component appears to behave pathologically, exploiting the very defensive mechanism meant to protect fluent speech. In doing so, it triggers the very outcome the system is trying to avoid: stuttering.

### 3.5 Neural mechanisms of anticipation and error monitoring of stuttering

To understand who these recruited components are, it is first essential to define the key regions that constitute the SMS itself. Research consistently identifies the anterior cingulate cortex (ACC) (Nozari, 2025; Nozari et al., 2011) as a central and highly sensitive hub for self-monitoring, responsible for detecting conflicts and errors in ongoing behavior. In parallel, the lateral prefrontal cortex facilitates processing by directing attention toward task-relevant stimuli, thereby enhancing stimulus–response mapping (Nozari et al., 2016; Nozari, 2025). Within this framework, the dorsal portion of the right lateral prefrontal cortex (R-DLPFC) plays a crucial role in anticipatory processes, supporting planning and expectation in speech production (Jackson et al., 2022).

From this standpoint, the aim is to examine the functional state of these two regions in PWS, as well as to identify other brain areas that are closely linked to self-monitoring processes and that, at the same time, exhibit abnormal activity or development in this population.

Research by Jackson et al. (2022) highlights a network of right hemisphere regions that play central roles in the anticipation and monitoring of stuttering. The R-DLPFC emerges as a key node within this system. It becomes hyperactive prior to the onset of speech when a person expects to stutter, reflecting the brain’s attempt to predict errors and apply cognitive control. Closely linked to this process is the ACC, which contributes to emotion regulation, decision-making, error detection, attention, and conflict monitoring. The ACC provides error signals to prefrontal regions, bridging emotional and cognitive responses during anticipated dysfluency. The R-DLPFC is part of the Frontoparietal Network (FPN) and works in coordination with the right supramarginal gyrus (R-SMG). However, under stuttering anticipation, connectivity between the R-DLPFC and R-SMG decreases, suggesting that anticipation may disrupt the stability of the network responsible for supporting fluent speech.

In line with these findings, Toyomura et al. (2018) provided compelling evidence that emotional circuits play a direct role in the expression of stuttering during real-life communication. Their findings showed that activity in the right amygdala was positively correlated with both the number of stuttering episodes and the level of emotional discomfort (as measured by SUD scores) during interpersonal speech tasks involving eye contact. This was the first study to directly show that amygdala activation tracks actual speech disfluencies in PWS during live communication, rather than reflecting only generalized or trait anxiety. In parallel, reduced activity has been reported in the medial prefrontal cortex in PWS. Notably, the ventromedial prefrontal cortex (vmPFC), a key subdivision of this region, plays an essential role in regulating amygdala-driven emotional responses. Findings of abnormal dopaminergic signaling in the vmPFC indicate a possible functional alteration in this regulatory pathway (Wu et al., 1997). This suggests that reduced prefrontal control fails to inhibit amygdala overactivation, allowing fear and negative emotional memories to influence ongoing speech. Toyomura et al. (2018) also reported increased activation in the right insula (R-insula) during speech tasks in PWS, a region critically involved in salience detection, interoceptive awareness, and the integration of emotional and cognitive signals relevant to self-monitoring and anticipatory control.

Together, these findings suggest that stuttering involves an interaction between the error monitoring networks (R-DLPFC, ACC), the integration region (R-SMG), and emotional circuits (amygdala, vmPFC, and R-insula).

### 3.6 Mapping the self-monitoring system in PWS

To hypothesize how the SMS operates in PWS, it is first necessary to consider its function in fluent speakers. Two scenarios can be outlined. In the first scenario, where social evaluative pressure is absent and there is no conscious focus on speech, both fluent speakers and PWS exhibit similar SMS activity. In this context, the ACC detects error signals in speech production regions in PWS and conflict or competition signals in fluent speakers. These signals are sent from the ACC to the lateral prefrontal cortex (LPFC), which is responsible for making adjustments based on this monitoring to optimize the production process. This process occurs entirely at a subconscious level.

The second scenario highlights the critical differences. In the presence of social evaluation and the absence of distracting stimuli, conscious attention is directed toward speech.

Social evaluation is determined by higher-order cognitive regions, which engage the amygdala to assess threat-related significance and retrieve prior memories of similar socially evaluative events. In parallel, the right insula contributes to monitoring self-awareness and reflecting on interoceptive bodily sensations associated with social stress. Once a socially evaluative context is established, these higher cortical regions, together with the amygdala and insula, interact with the SMS to mediate the transition from subconscious, automatic speech error monitoring to conscious error detection. Following this transition, the SMS amplifies error-related signals as warning signals, prompting the recruitment of additional neural resources to support speech production. Among the key regions involved in this compensatory process are control, inhibitory, and conflict-monitoring regions, particularly the pre-supplementary motor area (pre-SMA) and the right inferior frontal gyrus (rIFG) (Aron et al., 2016).

Fluent speakers in such situations, when exposed to social evaluation and following the shift of self-monitoring from a subconscious to a conscious process, recruit both the pre-SMA and the rIFG to support speech production. Under these conditions, the pre-SMA plays a particularly prominent role, as it is involved in processing conflict signals and engaging inhibitory control mechanisms, which can slow or delay speech output until the conflict is resolved (Aron et al., 2016).

In PWS, under such socially demanding situations, the process begins with the presence of clear error signals within the auditory–speech–motor systems. Although these signals alone are not sufficient to cause stuttering, they are highly salient. Consequently, the SMS becomes overactive in PWS (Arnstein et al., 2011; Arenas, 2017), a response that aligns with its fundamental role in error monitoring.

The persistence of these error signals leads to increasingly intense engagement of the SMS, thereby amplifying its overall activity. This heightened engagement becomes particularly evident during the anticipation phase, when the speaker prepares for upcoming speech, such as during a lecture, classroom discussion, meeting, or job presentation.

At the same time, higher-order cognitive regions, together with the amygdala and the insula, evaluate the social situation. The amygdala, in particular, appears to be overactive in PWS, likely due to repeated negative social experiences such as embarrassment and perceived social failure. These experiences condition the amygdala to interpret social situations as threats to personal value and social identity. This process, in turn, contributes to marked hyperactivity in the right insula, a region critically involved in self-awareness and the monitoring of bodily sensations.

These limbic and higher-order cognitive structures interact with the SMS to mediate a transition toward conscious error monitoring, even before speech initiation. This transition triggers the anticipation process, which is largely guided by the right dorsolateral prefrontal cortex (R-DLPFC), along with the coordinated involvement of the ACC, amygdala, insula, and other higher cognitive regions. Together, these areas work to identify the most important words in the upcoming utterance, as well as those perceived as most difficult. PWS can recognize “fear words” likely to provoke stuttering even before speaking (Arenas, 2012; Jackson et al., 2015; Jackson et al., 2018).

Through this interaction, warning signals are generated and distributed across the system. Importantly, these warning signals are not global, but rather specifically tied to particular words that the system has identified as critical or threatening. While the emotional responses produced by the amygdala and associated regions are relatively general (e.g., anxiety and tension), the warning signals themselves become sharply focused on the specific word that the speaker is about to produce, the word perceived as most difficult and accompanied by a pronounced increase in amygdala activity and emotional arousal.

In this state, the SMS recruits additional neural regions for support, most prominently the pre-SMA and the rIFG. However, despite this compensatory recruitment, the system ultimately fails to stabilize fluent speech, and stuttering emerges. This outcome suggests that at least one of the recruited regions, rather than facilitating fluent production in a normal manner, becomes maladaptively involved in the emergence of the stuttering behavior itself.

During stuttering events, the same mechanism occurs but at a much faster pace. Considering that there is no specific threshold in speech production areas for the onset of stuttering, the question arises: which structure is responsible for triggering it? The pre-SMA is an intuitive candidate; however, it primarily responds to conflict signals rather than warning signals (Aron et al., 2016). Warning signals, in contrast, are not conflict-based; they are alerting signals that recruit other regions to facilitate fluent speech. Conflict signals may be minor and processed subconsciously, or major and represent hesitation between options, which is not the case in stuttering. Hesitation in PWS is typically a consequence, not a cause, such as selecting an alternative word when a target word is difficult. PWS demonstrate linguistic flexibility, attempting different speech initiations, using preambles, or substituting words. Therefore, stuttering does not reflect conflict but rather a genuine inability to produce a specific word.

Another factor that may contribute is the weakened connectivity between the R-DLPFC and the right supramarginal gyrus (R-SMG). This disruption could result from situational pressure (fear and stress), particularly amygdala-driven, with potential modulation deficits in the vmPFC, which normally regulates amygdala activity. Alternatively, this weakened R-DLPFC–R-SMG connectivity may itself represent the actual warning signal elevated by the system. This leads to a secondary hypothesis: the lateral PFC may fail under pressure to correct speech. However, this mechanism has only been reported once and requires further investigation. Moreover, fluent speech remains possible when a difficult word is replaced by another, suggesting that the involvement of R-DLPFC–R-SMG disruption may be unlikely. If situational pressure were the primary factor causing disrupted connectivity between these regions or reducing the threshold for the onset of stuttering, pharmacological interventions aimed at reducing anxiety and tension would be expected to exert a strong and consistent effect. Contrary to this expectation, such interventions have not demonstrated substantial efficacy (Molt, 1998). In contrast, the mere cognitive acceptance of stuttering, acknowledging it and adopting a mindset of living with it, has been shown to reduce stuttering severity to some extent (Boyle, 2011; Moreno-Jiménez et al., 2021; Israel et al., 2023). This effect appears to arise primarily through two mechanisms: first, by decreasing conscious focus on stuttering, and second, by alleviating the social pressure associated with self-acceptance. Together, these processes may contribute to a reduction in the generation and dissemination of warning signals by the SMS. Thus, stuttering appears to arise from a mechanism beyond general anxiety or tension.

When these structures are examined closely (SMS, amygdala, and insula), they represent a fundamentally normal and adaptive response of systems responsible for self-monitoring. There is, therefore, nothing inherently abnormal or pathological in this process. Even in cases of overactive self-monitoring in PWS (Arnstein et al., 2011; Arenas, 2017), the presence of error signals within speech-related regions compels this system to increase its sensitivity toward those signals. Such hyper-responsiveness/activity should be understood as a compensatory reaction, an attempt by the system to manage these error signals by recruiting regions with higher analytical and regulatory capacities. The overactivity of the amygdala can be explained in a similar manner.

Postma and Kolk (1992) showed no significant differences between PWS and those who do not in error detection accuracy, speed, or false alarm rates during self-produced speech with normal or masked auditory feedback. However, PWS detected fewer errors in speech produced by others, suggesting a potential phonological issue.

## 4 A dominant neural contributor to the emergence of stuttering

From this perspective, both the speech production regions and the SMS appear, according to our hypothesis, to be largely not directly responsible for the generation of stuttering symptoms.

Identifying the specific structure responsible for stuttering symptoms requires defining the necessary conditions that indicate it as the origin of stuttering. These conditions form the criteria for investigating its neural basis. Such a structure must satisfy four critical conditions. First, it must be strongly and functionally connected to speech production regions. Second, it must be directly linked to the SMS and capable of receiving its signals. Third, this structure must exhibit a pronounced sensitivity to the SMS’s warning signals, such that it exploits the heightened monitoring state to actively suppress speech, specifically targeting the words and utterances that the SMS has flagged for increased scrutiny. Finally, this structure must demonstrate atypical development or functional alterations beginning early in childhood, coinciding with the onset of the disorder.

The region that most convincingly fulfills all of these conditions is the right inferior frontal gyrus (rIFG).

### 4.1 Beyond self-monitoring and speech production: the rIFG in stuttering symptom expression

Anatomically, the rIFG is part of the right prefrontal cortex and is widely implicated in inhibitory control and response suppression as a key component of right-lateralized control networks (Aron et al., 2004, 2014). However, converging evidence indicates that the rIFG is a key component of the inhibitory network and that it operates within a broader distributed system rather than serving as its singular central locus (Hampshire et al., 2010; Choo et al., 2022). In addition, the rIFG also supports broader functions beyond inhibition. For example, a voxel-wise meta-analysis of temporal processing identified the rIFG (with SMA) as a consistent component across timing conditions, motivating its inclusion in a putative “core timing network” (Wiener et al., 2010). In addition, Hampshire et al. (2009) demonstrated tight target tuning in the rIFG, showing selective responding to the currently defined target over distractors, consistent with a role in goal-dependent selection rather than inhibition alone. In line with this broader framing, the present literature implicates the rIFG in executive functions such as adaptive attentional control, target selection, attentional switching, and memory-related control and retrieval (Duncan and Owen, 2000; Wagner et al., 2001; Anderson et al., 2004; Hon et al., 2006; Hampshire et al., 2007, 2008, 2010). Some recent studies have started linking the rIFG to depression (Rolls et al., 2020).

This functional diversity has motivated accounts linking the rIFG to SMS, and in the literature, it is often discussed in relation to the cognitive control network and the behavioral inhibition system (BIS) (Usler, 2022). Importantly, the rIFG is not only embedded in multiple cortical networks, but it also shows principled connectivity with speech-relevant cortico–basal ganglia circuits via two pathways: an indirect cortico-striatal pathway (rIFG→caudate/striatum, expressed through basal ganglia channel dynamics) and a fast hyperdirect pathway (HDP) linking the rIFG to STN, often framed as a global brake/broad pause mechanism (Jahfari et al., 2011; Aron et al., 2016; Chen et al., 2020).

While a consistent body of research, including studies by Chang et al. (2008, 2015, 2019) and Chow et al. (2023), has not consistently identified significant abnormalities in the development of gray and white matter in the rIFG in CWS, suggesting **relatively preserved** structural development, Beal et al. (2013) suggested that reduced gray matter volume in the rIFG may be present, particularly in relation to greater stuttering severity; however, what appears to be more affected is the **connectivity** of this area. Specifically, Neef et al. (2023) reported that the pars opercularis (the most prominent part of the rIFG, which plays a crucial role in functions related to executive control, language processing, and inhibition) in CWS aged 3–11 shows a mixed connectivity profile, with enhanced coupling to insula and somatomotor regions implicated in motor control and inhibition, alongside weaker coupling with components of the dorsal attention network, which supports attentional and top–down cognitive regulation.

The weaker coupling of the rIFG with components of the dorsal attention network suggests a concerning implication: the rIFG’s impaired connection with these areas may lead to difficulty in processing warning signals, particularly those associated with language and speech processing, as indicated by the signals highlighted in the SMS. Instead of engaging with warning signals specifically related to speech and language, the rIFG, specifically the pars opercularis, appears to struggle with understanding or responding to these signals. Given that the same region shows enhanced coupling to the insula and somatomotor regions implicated in motor control and inhibition, this indicates a greater tendency toward inhibitory functions. When combined with the failure to properly process warning signals from the SMS, the rIFG seems to initiate a direct inhibitory response to the speech production areas, leading to the mechanism of blocking/freezing of speech (Aron et al., 2016; Usler, 2022). At the same time, adaptive attentional control, target selection, inhibition initiation processes, attentional switching, performance in the timing network, and memory-related control and retrieval seem to function well in most tasks. The weakness appears to be specific and limited to speech-related signals, particularly the warning signals from the SMS. Otherwise, if there were a real defect in this region, we would expect to observe greater effects, such as difficulty initiating and stopping actions (Sundby et al., 2021; Choo et al., 2022). However, this is not the case (Wiltshire et al., 2025). If this assumption holds, the question arises: how would the rIFG behave in PWS if its development in most of its functions is normal? In this scenario, we would need a model where the rIFG is functioning normally without any issues, while simultaneously, there is a problem with speech or speech production areas.

A clear example of such a scenario can be found in patients who have suffered a stroke affecting the speech production areas. In these patients, it is assumed that their rIFG remains completely intact. As reported by Winhuisen et al. (2007), in patients with severe damage to left-sided language areas, the rIFG becomes more activated, contributing to language recovery, especially in cases of aphasia (language deficits). Similarly, van Oers et al. (2010) suggest that the rIFG plays a compensatory role in language recovery after stroke, particularly by supporting non-linguistic cognitive processing. Additionally, a meta-analysis by LaCroix et al. (2021) shows that in post-stroke aphasia, the rIFG plays a compensatory role in language production, with increased activation in the right frontal and temporal cortices.

This is exactly what we suggest the rIFG attempts to do in PWS. When the error signals are detected by the SMS under social evaluation pressure and conscious error monitoring, the system responds to them as warning signals. The rIFG then joins the SMS and performs its functions of attention and language support through its multiple roles, so both the rIFG and the SMS work together as compensatory or defensive mechanisms, striving to ensure the most accurate speech production possible. The only difference here is that PWS seem to have subtle dysfunctions in the connections within this region, causing the rIFG’s compensatory mechanism to become, in some cases, the primary mechanism that contributes to the manifestation of stuttering.

### 4.2 When compensation becomes the cause: the rIFG in stuttering expression

The mechanism underlying this process can be understood as follows: as discussed earlier, abnormal connectivity within the rIFG makes it more susceptible to warning signals generated by the SMS, leading to increased inhibition. This phenomenon, which we refer to as rIFG oversensitivity, suggests that due to these connectivity issues, the rIFG becomes excessively responsive to speech-related warning signals produced by the SMS. This heightened sensitivity can contribute to dysregulation in speech production, as the rIFG becomes overly reactive to signals that would normally help regulate speech output.

Supporting the involvement of the rIFG in stuttering, meta-analytic fMRI studies have consistently reported excessive recruitment of right frontal regions, particularly the frontal/rolandic operculum and anterior insula, extending into the inferior frontal gyrus in PWS compared with fluent speakers (Budde et al., 2014; Belyk et al., 2015).

Yang et al. (2016) showed that stronger rs-fMRI coupling between the right IFG and right cerebellar lobule VI is associated with greater stuttering severity, while Ghaderi et al. (2018) reported increased resting-state EEG network centrality of the right inferior frontal cortex in PWS compared with fluent controls.

In line with this interpretation, Neef et al. (2018) reported that the posterior rIFG (pars opercularis) in PWS shows increased structural coupling with pre-SMA/SMA and descending fibers traversing basal ganglia territories toward the STN, with the strength of these pathways scaling with stuttering severity. Within their framework, such enhanced involvement of the rIFG–STN inhibitory circuitry may disrupt the smooth sequencing of speech motor programs, offering convergent evidence that excessive recruitment of global suppression mechanisms can contribute to the instability observed during speech production.

The phenomenon of rIFG oversensitivity can explain why stuttering occurs with certain words in a sentence, while others are produced smoothly. The inhibition signal from the rIFG appears to be tied to specific words highlighted by the SMS, while other words in the sentence pass through without interruption. The inhibition is thus directly linked to the rIFG’s difficulty in properly understanding and responding to these warning signals. This difficulty may also lead to the over-engagement of the rIFG with the warning signals, where the area attempts to process and focus on them more intensely. This heightened focus may increase tension within the region, prompting an inhibitory response. Therefore, the inability to understand the signal can be viewed as a broad concept, encompassing not only the failure to process the signal but also the difficulty in interpreting it or the abnormal engagement with it. All of these factors contribute to an increase in tension and conflict within the region, making the signal appear as something dangerous that requires inhibition. This situation specifically leads to the SMS itself being perceived as the cause of stuttering (Arenas, 2017). However, according to our hypothesis, the rIFG, particularly the pars opercularis, acts as a hidden mechanism that underlies these symptoms, ultimately driving the overt stuttering behaviors (Neef et al., 2018).

This raises another question: in the absence of warning signals, how would the rIFG behave?

In the absence of warning signals, the rIFG would likely function normally, similar to other regions of the brain. It could also carry out its typical role in the compensatory mechanism, helping to maintain normal speech production and cognitive processes when there is impairment in those regions (Winhuisen et al., 2007). In line with this, Wiltshire et al. (2025) found no evidence of globally heightened rIFG activation in a generic stop–signal paradigm, suggesting that PWS do not appear to exhibit a universally overactive, domain-general inhibition system during non-speech tasks. This finding is expected, as the region does not appear to demonstrate abnormal development sufficient to be considered a primary dysfunction.

Thus, the activity observed in this area during choral reading in PWS is not a coincidence and should be interpreted as a compensatory mechanism (Toyomura et al., 2011; Etchell et al., 2014), as it highlights that the rIFG continues to perform its core functions in tasks involving core timing networks, attention, and memory retrieval in the absence of warning signals. However, it is only in stuttering contexts that this area shows abnormal activity. Under fluency-inducing conditions, where the SMS is primarily engaged by external stimuli (such as choral reading and singing), and in situations where error signals in the speech areas do not transform into warning signals (e.g., talking to oneself or speaking alone), along with non-verbal, non-speech tasks—even those inherently involving inhibitory actions—the rIFG will perform its typical functions. It may either compensate by working with the SMS according to the context or execute its normal functions as needed.

Supporting this notion, Connally et al. (2018) examined inferior frontal activity using two complementary approaches: a group-level comparison restricted to fluent utterances and a within-speaker state analysis contrasting dysfluent and fluent speech. Their findings revealed no overactivity in the fluent-only group comparison. However, the state analysis demonstrated increased activation of the right inferior frontal cortex, extending into the opercular cortex and anterior insula, during dysfluent speech relative to fluent speech within the same individuals. Notably, these right frontal regions were not overactive during fluent speech in PWS compared to controls, and no regions showed greater activation during fluent than dysfluent speech. Based on these results, Connally et al. (2018) interpret right hemisphere overactivity as reflecting an inhibitory or “stopping” response that emerges primarily during dysfluent states. Similar to this conclusion, the rIFG has also been discussed as a main area in the causality of stuttering by Usler (2022).

In the study conducted by Preibisch et al. (2003), unlike the findings in Connally et al. (2018) which suggested normal function of the rIFG without any overactivity in fluent speech in PWS compared to controls, Preibisch observed overactivity in the right frontal operculum (RFO) during fluent speech in PWS relative to controls. To consider this case as well, the explanation is straightforward: according to our hypothesis, as long as there is fluent speech in PWS, this implies that warning signals are not represented. When warning signals are absent, the RFO will generally perform its typical function, whether through normal activity (as observed in the 2018 experiment by Connally et al. 2018) or overactivity as a compensatory response of this region in conjunction with the SMS.

In Preibisch et al. (2003) second experiment, where participants performed a silent synonym judgment, normal activity was observed in the RFO. This finding is also consistent, as the task did not require speech or stuttering, allowing the region to function in its typical, unaltered manner.

### 4.3 The rIFG at the crossroads of compensation and causation in stuttering

Based on the research literature, it is unclear whether the rIFG acts as a compensatory mechanism or plays a causal role in stuttering. What we propose is that it serves both functions. Given that this region has developed normally, it carries out its natural functions. However, when issues arise in speech production areas, such as in various neurological disorders, the rIFG seems to play a compensatory role (Winhuisen et al., 2007; van Oers et al., 2010; LaCroix et al., 2021). This compensatory mechanism is also active in stuttering, but due to a subtle dysfunction in the connectivity between the pars opercularis and its attention networks, as well as increased connectivity with inhibition and motor control networks, this shift in connectivity results in the rIFG transitioning from a compensatory or natural role to a primary cause of stuttering.

The key factor in this shift is the warning signals generated by the SMS. These signals trigger the rIFG’s pathological effects, leading to speech inhibition. The underlying connectivity disruption makes the rIFG excessively sensitive to speech-related warning signals, causing it to inhibit speech production and directly contribute to stuttering.

We propose a shift in perspective here: rather than locating the critical threshold for stuttering within speech production regions, we argue that the primary threshold governing the onset of stuttering resides in the rIFG itself. Altered connectivity, characterized by reduced coupling with attentional networks and enhanced coupling with inhibitory–motor networks, appears to lower the inhibitory activation threshold of the rIFG, rendering it unusually sensitive to warning signals originating from the SMS. Importantly, this heightened sensitivity seems to be specific to speech-related processes. In the absence or attenuation of such warning signals, the rIFG reverts to its canonical role, engaging cooperatively with the SMS in a compensatory manner.

This clearly explains why stuttering occurs on certain words but not on others, and why PWS often can substitute problematic words. Collectively, these observations suggest that speech production regions operate adequately to enable fluent speech in most situations. In contrast, the rIFG appears to intervene selectively when words are perceived as significant, temporarily halting or disrupting speech. Once such a word is replaced, the speech production system resumes its operation, effectively bypassing the inhibitory signal generated by the rIFG, which was specific to that particular word rather than to speech production as a whole.

The core of the issue lies in the warning signals generated by the SMS. These signals are key to understanding the situational variability and explaining the rIFG’s response within the context of our hypothesis.

Despite the complexity of this phenomenon, it is not beyond the reach of current methodologies. The mechanisms at play can be practically measured and accurately assessed using available tools, and we will explore these further in the Empirical Tests and Falsifiable Predictions section.

After reviewing all this information and reaching this point, an important question arises: If the rIFG causes stuttering through the initiation of an inhibitory process, on which pathway of the rIFG does this initiation occur, and how?

## 5 Pathway-specific inhibitory mechanisms of the rIFG in stuttering

During the developmental window in which stuttering typically emerges (approximately 2–5 years of age) and throughout the subsequent period up to around 8 years, any functional influence of the rIFG is more plausibly expressed via the indirect (fronto-striatal) pathway, given the evidence that the HDP undergoes substantial maturation later, between 9 and 12 years of age (Cai et al., 2019). Within this earlier stage, the stuttering profile tends to exhibit a set of distinctive characteristics; the disfluencies generally appear as sound, syllable, or word repetitions, while prolongations are less frequent and blocking is uncommon.

A study by Kim et al. (2020) investigated speech–auditory–motor learning deficits in CWS. The findings revealed that both children and AWS exhibit significant impairments in adapting to altered auditory feedback. Younger children (3–6 years) showed the most severe deficits, while older children (7–9 years) demonstrated some improvement but never reached the adaptation levels of their nonstuttering peers. These results suggest that impaired speech–auditory–motor learning is a fundamental factor in the onset and persistence of stuttering, and continues into adulthood (Daliri and Max, 2018).

Abnormalities in the striatum and the basal ganglia–thalamocortical (BGTC) loop (Chow et al., 2023), along with deficits in auditory–motor learning, are key factors in repetition-dominant dysfluency. These factors may be further influenced by input from the rIFG within the indirect pathway.

Studies show that CWS exhibit higher sympathetic arousal, greater skin conductance, and smaller blood pulse volume amplitudes (Walsh and Usler, 2019). CWS also tend to have lower IQ scores compared to control groups, although some perform within the normal range (Chow et al., 2023). Additionally, their phonological working memory and attention are impaired (Anderson and Wagovich, 2010; Eichorn et al., 2018). CWS may face difficulties in cognitive flexibility, especially during childhood (ages 2–12), but differences may not be evident in younger children due to immature executive functions or evolving stuttering mechanisms (Paphiti and Eggers, 2022). Some CWS also exhibit hyperactivity and compulsive behaviors (Alm, 2014). These conditions vary across individuals and can be considered risk factors influenced by the disorder. These impairments indicate significant physiological factors that profoundly impact various brain functions. These factors will be further explored in the physiological section of the paper.

Variability in stuttering severity remains largely present during childhood, indicating that it is a trait present from the onset of this disorder (Rasoli Jokar et al., 2025). In CWS, the influence of the SMS differs from that observed in adults. As discussed earlier, rather than selectively focusing on critical words, feared words, and the most important words within a sentence (Bloodstein, 1955; Kaasin and Bjerkan, 1982; Usler, 2022), the SMS in CWS may operate through different patterns, such as increased communicative or environmental pressure, which in turn precipitates stuttering. Consistent with this view, Howell et al. (1999) demonstrated that stuttering in ages 2–6 years occurs more frequently on function words (words that serve a grammatical purpose rather than carry specific meaning, such as pronouns, prepositions, and conjunctions). However, as they get older, their stuttering tends to shift to content words (words that carry meaning in a sentence, such as nouns, verbs, adjectives, and adverbs).

### 5.1 The critical period in the development of persistent stuttering

The evidence from preschool-aged children provides valuable insights into stuttering at this developmental stage. However, the disorder does not remain static across development. As children grow older, particularly between the ages of 9 and 12, the same period during which the HDP undergoes substantial maturation, the behavioral and linguistic profile of stuttering begins to transform. The disorder that initially manifests through mild sound or syllable repetitions in preschool years often shifts toward more severe blocks and prolongations, and the overall stuttering pattern increasingly resembles that observed in adults with persistent stuttering (Howell et al., 2008). Concurrently, disfluencies transition from function words to content words (Howell et al., 1999). This developmental shift marks a potentially sensitive phase in the trajectory of the disorder. As noted by Singer et al. (2020), late onset has been associated with a higher likelihood of persistence compared to earlier onsets. Importantly, the severity of stuttering around age eight has been shown to predict later outcomes with approximately 80% accuracy (Howell and Davis, 2011), suggesting that children entering this period with high stuttering severity are significantly less likely to recover spontaneously.

Notably, this period also coincides with a marked shift in sex ratio. While the prevalence of stuttering in early childhood is relatively balanced between males and females, longitudinal data show a sharp divergence beginning around age nine, widening from approximately 2:1 in the preschool years to about a 4:1 male-to-female ratio by age nine (Dworzynski et al., 2007). This growing disparity aligns with the same developmental window in which recovery rates decline and symptom patterns intensify, reinforcing the notion that this stage represents a critical juncture in the maturation of stuttering.

Several studies have also reported the onset of stuttering symptoms during this age window (Boyce et al., 2022), with a notable cluster around age ten (Howell et al., 2008), referencing data from Andrews and Harris (1964). However, these findings must be interpreted cautiously, as many rely on retrospective parental reports or self-assessments rather than direct clinical observation, factors that may inflate or distort the true incidence. Moreover, most modern investigations have been biased toward early childhood samples, typically following children only until age six or eight. As noted by Yairi and Ambrose (2013), “samples of children from birth to age six will miss some later onsets,” and “most studies cease follow-up below age eight, thus excluding a proportion of later onsets.” Consequently, the 9–12 age range remains underrepresented in contemporary research, leaving an important developmental period largely unexplored.

Nevertheless, even the limited evidence available raises intriguing possibilities. The combination of robust behavioral changes, such as the shift in disfluency type, the widening gender ratio, and the decline in recovery probability, along with preliminary evidence of new onsets during this period, suggests that late childhood may represent a significant turning point in the evolution of stuttering.

Because this developmental period coincides with the maturation of the HDP, which has been identified as a key route linking the rIFG with the STN, it becomes necessary to examine this pathway in greater depth and with greater specificity.

### 5.2 The HDP as a critical developmental pathway in stuttering

The HDP is a fast frontal cortex-to-STN projection that enables prefrontal control regions to rapidly influence basal ganglia output. In this view, the HDP supports two related control circuits. First, a stopping circuit, in which the rIFG (and potentially the pre-SMA) engages the STN via the HDP to implement rapid suppression of an initiated response. Second, a conflict circuit, in which dorsomedial frontal regions (pre-SMA/dmPFC) recruit the STN via the same HDP to impose a brief delay when competing response tendencies are co-activated, thereby increasing inhibitory basal ganglia output and raising the decision threshold before committing to an action. Together, these circuits describe how HDP-mediated frontal input to the STN can contribute to both action cancellation (stopping) and response slowing under competition (conflict) (Aron et al., 2016).

In the context of motor inhibition, Chen et al. (2020) explained that the HDP acts as a rapid means for stopping actions, such as when a person needs to cancel a planned movement or response due to changing environmental demands. This pathway is significantly faster than the more traditional indirect and direct pathways within the basal ganglia (Nambu et al., 2002).

As previously discussed, stuttering occurs particularly due to an abnormal over-reaction of the rIFG. This over-reaction is derived from excessive sensitivity to warning signals from the SMS and the amygdala, which rapidly initiates an inhibitory process causing an excitatory signal to pass through the HDP to the STN. This results in a complete freeze and sudden halting of the entire speech production system (Neef et al., 2018; Usler, 2022) until a signal with minimal warnings can pass through, such as switching from the intended word to a less suitable one that does not trigger the attention of the SMS.

Through the HDP, the rIFG is anatomically positioned to transmit rapid, global inhibitory signals to the speech motor system—a capacity that can, in principle, disrupt the continuity of articulatory programs and produce the kind of speech “freezing” observed in stuttering. Developmental evidence indicates that this pathway undergoes substantial maturation between 9 and 12 years of age (Cai et al., 2019), suggesting that its emerging inhibitory efficiency during this period may plausibly contribute to the clinical transition often noted in this age range. As the HDP matures, the rIFG becomes increasingly capable of exerting fast, system-level inhibitory control over speech.

An alternative interpretation must also be acknowledged: the marked clinical shifts observed between ages 9 and 12 may reflect developmental forces that are psychological rather than neurobiological in nature. During this period, children undergo a well-documented expansion in metacognitive capacity, social self-awareness, and emotional sophistication. These normative maturational processes could, in principle, amplify the salience of communicative demands, sharpen sensitivity to listener evaluation, and heighten self-monitoring during speech, each of which is known to modulate stuttering severity. Under this account, the transformation of stuttering from predominantly repetitions to more effortful blocks could emerge without invoking any specific neural reorganization. In this scenario, the temporal correspondence between symptom change and HDP maturation may represent a developmental coincidence rather than a mechanistic link.

However, if the HDP does contribute causally to these changes as proposed in the present framework, this would offer a unified and mechanistically coherent explanation for several long-standing clinical observations, including the shift from repetitions to blocks, the emergence of true speech “freezing,” the sharp decline in recovery after age 12, and even the well-documented sex ratio differences in recovery from stuttering. This possibility opens an important avenue for theoretical refinement and empirical investigation.

#### 5.2.1 From repetitions to blocks

In early childhood, stuttering usually appears in the form of repetitions. These patterns are possibly driven by errors in the direct and indirect pathways of the striatum, facilitated by abnormal auditory–motor integration, and their severity fluctuates across situations due to modulation by the SMS. During this stage, the rIFG already sends inputs to the striatum, contributing to stuttering as part of the direct and indirect pathways.

Between 9 and 12 years of age, a critical developmental transition may occur with the maturation of the HDP. This maturation introduces a rapid and potent route through which the rIFG and pre-SMA can exert inhibitory control more directly. Through this pathway, inhibitory signals can be transmitted to the speech motor system without obligatory mediation by the striatum. When engaged by warning signals originating from the SMS, this mechanism may plausibly precipitate abrupt, system-level inhibition of speech motor programs, thereby contributing to a clinical shift from repetition-dominated disfluencies to more block-like interruptions.

#### 5.2.2 Onset of stuttering between 9 and 12 years

We propose that a subset of children may carry a latent vulnerability within speech–auditory–motor integration networks and the rIFGop, which remains clinically silent during early childhood because the neural mechanisms required to express it are not yet fully developed. With the maturation of the HDP between ages 9 and 12, this latent vulnerability gains access to a fast, high-gain inhibitory route capable of interrupting speech motor output. When additional recruitment is needed by the SMS, mild blocks may begin to emerge even in children who previously showed only subtle or inconsistent signs of difficulty.

Once these initial blocks become consciously perceived, and especially when they elicit fear, embarrassment, or anticipatory worry, the SMS becomes increasingly hypervigilant. The resulting warning signals place additional load on the already atypical rIFG, amplifying its activity through the HDP and thereby intensifying both the frequency and severity of stuttering episodes.

#### 5.2.3 No recovery after age 12

Howell et al. (2008), drawing on longitudinal data from Andrews and Harris (1964), reported that no child who continued to stutter beyond age 12 achieved natural recovery. Within the framework proposed in this paper, this developmental boundary may reflect a neurobiological transition rather than a purely behavioral one. We hypothesize that the maturation of the HDP during late childhood strengthens a fast, high-gain inhibitory link between the rIFG and the speech–motor system. Once this pathway reaches functional maturity, it may render the stuttering pattern more rigid and less amenable to spontaneous normalization.

#### 5.2.4 Sex differences in stuttering persistence

This phenomenon can also be interpreted coherently. Evidence from Neef et al. (2023) shows that boys, particularly near the onset of stuttering, are more susceptible to the abnormal connectivity of the rIFG that we have discussed, compared to girls. Complementary findings from Usler (2022) indicate that boys demonstrate a more protracted maturation of the basal ganglia and the corpus callosum compared to girls, alongside slower development of speech–motor coordination. Boys are also more likely to engage in “freezing-type” defensive behaviors associated with the behavioral inhibition system, whereas girls display greater cognitive flexibility and a wider range of adaptive responses. Most girls recover before the HDP fully matures (approximately 9–12 years) (Yairi and Ambrose, 2013), which, within this hypothesis, may prevent the emergence of the strong rIFG speech–motor inhibitory loop associated with persistent stuttering. Taken together, these maturational and neurodevelopmental differences may jointly contribute to the markedly higher persistence rates observed in males.

#### 5.2.5 Shift in stuttering from function words to content words

Finally, the developmental shift from stuttering on function words in early childhood to content words in later years, together with the increasing clarity and salience of situational variability, can be explained by the ongoing maturation of the SMS and higher-order cognitive regions. As these systems develop, stuttering becomes progressively more selective, shifting from a generalized sensitivity to pressure or stressful situations toward a greater dependence on specific lexical items (particularly those perceived as most important within the sentence) rather than on contextual stress alone, as reported by caregivers in Rasoli Jokar et al. (2025).

Recent evidence suggests that cerebellar lobule VI may represent an important node in the neural network implicated in stuttering, alongside the striatum and inferior frontal gyrus. Associations have been reported between stuttering severity and abnormal connectivity of the right lobule VI with the rIFG in adults (Yang et al., 2016; Ghaderi et al., 2018), as well as altered perfusion of the left lobule VI in children (Liu et al., 2024). Disrupted intra-cerebellar connectivity and atypical coupling with frontal and motor regions further suggest potential bilateral involvement (Yang et al., 2016). Collectively, these findings support the hypothesis that lobule VI may contribute to motor–executive disruption in stuttering, warranting further targeted investigation.

## 6 Our neurological model of stuttering

**Figure 1:**
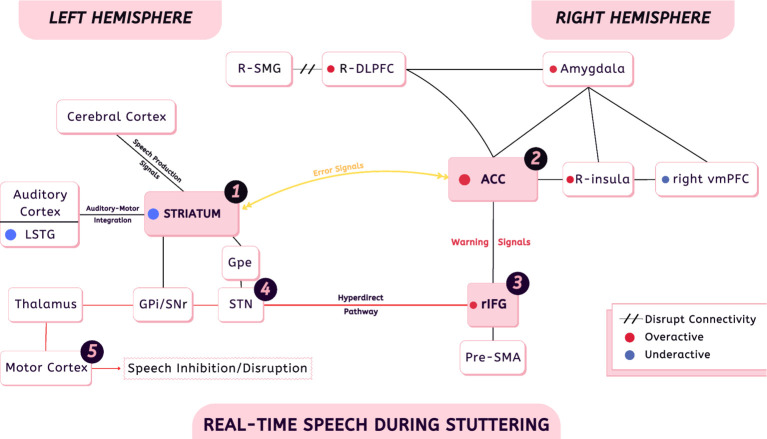
The process begins in the striatum when it receives integrated speech production signals from the cerebral cortex. These signals include motor plans, phonological encoding, cognitive intention, and emotional context. Although auditory predictions are also part of these inputs, the auditory cortex, particularly the left superior temporal gyrus (LSTG), is intentionally separated to emphasize its critical role in auditory-motor integration, a pathway strongly implicated in the pathophysiology of stuttering. Importantly, dysfunction within these speech-related neural pathways alone is not sufficient to produce stuttering. For stuttering to emerge according to our hypothesis, two additional factors are required: social evaluation and conscious speech error monitoring. Social evaluation is determined by higher-order cognitive regions, which engage the amygdala to assess threat-related significance and to retrieve prior memories of similar socially evaluative events. In parallel, the right insula contributes to monitoring self-awareness and reflecting interoceptive bodily sensations associated with social stress. Once a socially evaluative context is established, these higher cortical regions, together with the amygdala and insula, interact with the self-monitoring system (SMS) that is composed of the anterior cingulate cortex (ACC) and right dorsolateral prefrontal cortex (R-DLPFC) to mediate the transition from subconscious, automatic speech error monitoring to conscious error detection. Following this transition, the SMS amplifies error-related signals as warning signals. These warning signals do not emerge as isolated neural responses; rather, they represent the compressed output of continuous predictive, evaluative, and affective interactions among the ACC, R-DLPFC, amygdala, and right insula. Crucially, these signals are not globally distributed across speech output but are selectively tied to specific words identified as critical, threatening, or highly important by limbic and higher-order cognitive systems. Under heightened warning signaling, a particularly sensitive control region, the right inferior frontal gyrus (rIFG) detects this escalation. Due to its vulnerability to such signals, the rIFG, with or without the involvement of the pre-supplementary motor area (pre-SMA), rapidly engages the hyperdirect pathway by transmitting excitatory inputs to the subthalamic nucleus (STN). The STN excites GPi/SNr, which inhibit the thalamus more strongly → reducing thalamic excitation of the cortex → resulting in global motor inhibition (stopping/freezing of speech or movement). Importantly, this inhibition selectively targets specific words perceived as critical, feared, or highly important, while non-feared words will proceed through the motor system without suppression. Without activation of the hyperdirect pathway, speech would generally flow smoothly, with only occasional minor interruptions, primarily observed in children. The hypoactivity of the ventromedial prefrontal cortex (vmPFC) in this context may compromise its capacity to exert effective top–down regulation over amygdala hyperactivity. This failure of emotional regulation increases affective pressure within the self-monitoring network, particularly involving the ACC and R-DLPFC. Such elevated emotional load may account for the disruptions in functional connectivity observed during speech anticipation, especially between the R-DLPFC and the right supramarginal gyrus (R-SMG). globus pallidus internus (*GPi*) and externus (*GPe*), Substantia Nigra, pars reticulata (*SNr*).

The hypothesis presents a model representing a comprehensive integrative framework for how stuttering occurs in real-time speech (see Figure 1).

The diagram does not exactly portray the neural connections between these structures. Instead, it highlights the most important regions where these signals pass through or where these activities occur, presenting the diagram in a simplified and comprehensible format.

The term “hypersensitive,” previously used to describe abnormalities in the rIFG in stuttering, cannot be directly quantified with the available data. Therefore, we adopt the more operational term “overactive” in the model diagrams to indicate that this region is expected to exhibit disproportionately strong responses during stuttering, particularly during speech blocks.

In our model, hyperactive and hypoactive regions are treated as reflecting context-dependent physiological states rather than constant abnormalities. Specifically, the model aims to describe the behavior of these regions during stuttering. Outside of this context, the same regions may show different activity patterns, including during non-speech tasks, fluent speech, and fluency-enhancing conditions such as self-talk, singing, or choral reading. However, we also acknowledge that certain components of the proposed circuitry may remain abnormal even in the absence of overt stuttering episodes. This is supported by the work of Maguire et al. (2020), which demonstrates persistent striatal hypoactivity during both solo and choral reading, regardless of whether speech is fluent or dysfluent.

Although the present model was developed to explain stuttering, it can also be viewed as a simplified representation of speech production in fluent speakers. Speech production is a highly complex process involving multiple distributed cortical and subcortical regions. Accordingly, this framework does not aim to capture the full language network; rather, it focuses on those components most directly implicated in the emergence of stuttering. Classical language areas such as Broca’s and Wernicke’s regions were therefore not explicitly incorporated, as they are not considered primary drivers of stuttering initiation within the scope of the current hypothesis. From this constrained perspective, the model can be extended to fluent speakers as a normative system in which the same core components operate within typical functional ranges, while additional language regions contribute to the completion and refinement of speech generation. Importantly, this framework can also account for why stuttering-like disfluency may occasionally arise even in fluent speakers despite the absence of any prior history of stuttering. Such effects may be most apparent during public speaking, a context in which heightened stress and self-evaluative pressure have been shown to increase speech disfluencies even in non-stuttering speakers (Zhao, 2022; Sandoval et al., 2025). Under these conditions, enhanced activation of the SMS and particularly the amygdala component may give rise to excessive warning/conflict-related signals. If sufficiently amplified, these signals could engage the rIFG or the pre-SMA, leading to transient inhibitory or delay interference with speech output. Therefore, stuttering that may occur in fluent speakers originates solely from an emotional source. In contrast, in PWS, the cause is not solely emotional stress. Rather, emotional stress interacts with pre-existing dysfunction in speech production regions, which are interpreted by the SMS as error signals. When these error signals arise, such as in socially evaluative situations or when delivering an important message, they are perceived as warning signals. The emergence of such signals, combined with an oversensitive rIFG related to speech, leads to strong, repetitive, and involuntary speech interruptions.

Although this model is capable of explaining many phenomena associated with stuttering, it remains insufficient to fully account for certain types of individual variability, as well as variability over time. This limitation arises because the model is not yet complete; a final missing component remains, representing the crucial piece required for the model to explain most manifestations of stuttering. This component is hypothesized to reflect the primary underlying cause of stuttering. Accordingly, the following section focuses on this missing component by examining the neurophysiological basis of stuttering, proposed as a key element in completing the model.

## 7 The initial neural breakdown in stuttering development

### 7.1 Neurobiological alterations in developmental stuttering: an overview

Historically, through a long and continuous line of research, substantial progress has been made in identifying and characterizing the physiological alterations associated with stuttering, which collectively distinguish it as a unique neurobiological condition. Much of this research has traditionally focused on the development of gray and white matter. A considerable portion of these studies has converged on the finding that the BGTC is significantly affected by atypical development, particularly manifesting as reductions in gray matter volume, alongside disrupted white matter pathways extending across both hemispheres (Sommer et al., 2002; Guenther, 2006; Kell et al., 2024; Beal et al., 2013; Foundas et al., 2013; Connally et al., 2014; Civier et al., 2015; Chang et al., 2008, 2015, 2019; Chow et al., 2023).

However, the body of evidence extends well beyond structural abnormalities. In an integrative review of EEG and regional cerebral blood flow (rCBF) studies conducted in both children and AWS, Alm (2021) reported that PWS consistently exhibit reduced beta-band power during resting-state EEG. This pattern is commonly associated with diminished cerebral metabolic activity. In parallel, several rCBF studies have demonstrated reduced regional blood flow in frontal brain areas, particularly in regions characterized by high glycolytic demand, such as the inferior frontal gyrus (IFG).

Further evidence has emerged from neurochemical and metabolic imaging studies. An analysis of R2 relaxation maps in 41 AWS and 32 normally fluent controls revealed significant group differences in iron concentration, suggesting increased iron accumulation in the left putamen and left-hemisphere cortical regions critically involved in speech motor control (Cler et al., 2021).

Among these findings, one historically significant yet particularly striking study warrants special attention. A PET study by Wu et al. (1997) demonstrated a marked increase in 6-FDOPA uptake, indicative of elevated dopaminergic activity in several brain regions, including the right vmPFC, the left caudate tail, and limbic structures such as the deep orbital cortex, insular cortex, and extended amygdala in PWS.

Taken together, these findings portray the brains of PWS as systems characterized by widespread dysregulation, ranging from atypical gray and white matter development to iron accumulation in left-hemisphere structures, reduced beta oscillatory activity linked to cerebral metabolism, diminished regional cerebral blood flow, and, ultimately, pronounced dopaminergic hyperactivity across key neural circuits.

Within this apparent neurobiological heterogeneity, the critical question becomes whether a particular brain region emerges as a common convergence point for these abnormalities. Upon closer examination, the striatum stands out as the structure most consistently and profoundly affected.

### 7.2 The striatum as a convergence hub of neural dysregulation

The striatum is a major subcortical structure of the basal ganglia system, playing a crucial role in motor control, cognitive functions, action selection, and reward processing. It is primarily divided into two regions based on anatomical and functional distinctions: first, the dorsal striatum, which cosists of the caudate nucleus and putamen, is involved in motor coordination, procedural learning, and the modulation of voluntary movement. Second, the ventral striatum, which comprises the nucleus accumbens and olfactory tubercle, is part of the limbic system and plays a key role in reward processing, motivation, emotion, and reinforcement learning (Kandel et al., 2013).

The striatum was the earliest region to demonstrate atypical development, marked by reduced GMV (Chow et al., 2023). The extent of this reduction strongly correlates with stuttering severity during childhood. Brain imaging shows abnormally low activity in speech cortical areas and the striatum in PWS. However, when fluency is induced (solo vs. choral reading), cortical activity normalizes, but striatal activity remains low. Moreover, any increase in metabolism within this structure leads to noticeable improvements in fluency (Maguire et al., 2020). The striatum also showed the highest levels of iron accumulation (Cler et al., 2021) and contains the highest concentration of dopamine, up to three times greater than normal levels (Wu et al., 1997).

These findings demonstrate that the striatum stands out from other brain structures: it is the earliest region to show abnormalities and the one in which these alterations appear to persist most strongly. This observation raises an important question: why do the earliest detectable changes emerge in the striatum rather than in regions such as Broca’s area, Wernicke’s area, or the motor cortex? What characteristics make the striatum particularly susceptible to early disruption? As we argue below, the striatum possesses several features that distinguish it from other brain regions and may help explain this vulnerability.

The striatum is distinguished from most other brain regions by its exceptionally dense dopaminergic innervation. It receives substantial dopamine input from both the substantia nigra and the ventral tegmental area and expresses a high concentration of D1 and D2 type dopamine receptors, making it a primary target for dopaminergic signaling and supporting its central role in motor control, action selection, speech, motivation, and reward processing (Björklund and Dunnett, 2007; Delgado, 2007; Kreitzer and Malenka, 2008; Gerfen and Surmeier, 2011). This organization suggests that the striatum may be particularly sensitive to variations in dopamine levels. In line with this, a PET study by Wu et al. (1997) reported markedly elevated dopaminergic activity in specific striatal regions in PWS. These observations raise an important question: if striatal abnormalities emerge first, and given the close association between the striatum and dopamine, can dopamine dysregulation be considered the initial pathological event, or might other upstream changes precede and drive the observed dopaminergic alterations?

### 7.3 Circular causality in stuttering neurobiology

Here we enter a circular causal framework, in which each component can act both as a cause and a consequence of the others. Alterations in gray and white matter, metabolic activity, cerebral blood flow, iron accumulation, and dopaminergic signaling are not arranged in a simple linear hierarchy. Rather, each of these variables can influence the others bidirectionally, making it difficult to identify a single initiating event. In such a system, no element can be confidently labeled as the primary culprit; instead, each may simultaneously function as both origin and outcome.

One of the most influential hypotheses discussed within this context is the “disorder of energy supply to neurons” proposed by Alm (2021). This hypothesis argues that impaired metabolic support to neurons may represent the primary dysfunction from which many downstream abnormalities emerge. Alm extensively reviewed and analyzed experimental evidence suggesting that metabolic insufficiency could precede and drive changes in neural signaling, structure, and function. Another well-known hypothesis is lysosomal trafficking dysfunction, which proposes that impairments in lysosomal function lead to intracellular waste accumulation, subsequently triggering widespread cellular and neural dysfunction (Kang et al., 2010).

Within our own model, however, the central objective is to identify which of these variables possesses the greatest explanatory power—one that can simultaneously account for both the clinical manifestations of stuttering and the physiological and structural alterations observed in the brain. In other words, we are searching for a unifying thread capable of linking the neurophysiology of stuttering with its phenomenology. From this perspective, dopamine emerges as the most compelling candidate.

### 7.4 Dopamine as a unifying mechanism

Dopamine is one of the brain’s most influential neurotransmitters and neuromodulators, often described informally as the “molecule of life” and the “molecule of more” due to its unique and wide-ranging influence. In PWS, dopamine levels have been found to be abnormally elevated across several brain regions, including the medial prefrontal cortex, deep orbital cortex, insular cortex, extended amygdala, auditory cortex, and caudate tail. Notably, dopamine activity has been reported to reach nearly threefold higher levels in both the left caudate tail and the right vmPFC in PWS (Wu et al., 1997). Given dopamine’s critical role in regulating cognition, emotion, motivation, and motor control (Speranza et al., 2021), such elevations raise concerns that this increase is not merely a general symptom but may carry deeper significance.

Like the other variables discussed, dopamine is deeply embedded in reciprocal causal relationships with metabolism, cerebral blood flow, iron accumulation, and gray and white matter development. Dopamine has the capacity to modulate metabolic activity and, consequently, cerebral perfusion (Choi et al., 2006; Knutson and Gibbs, 2007; da Silva et al., 2011; Alm, 2021). It may act as a primary driver of iron accumulation in the left hemisphere (Hare and Double, 2016; Cler et al., 2021).

Importantly, the striatum is among the earliest regions to show abnormalities in developmental stuttering. This is particularly notable given that the striatum is one of the most dopamine-dependent structures and maintains direct connections with the two primary dopaminergic nuclei: the VTA and the substantia nigra.

**Figure 2:**
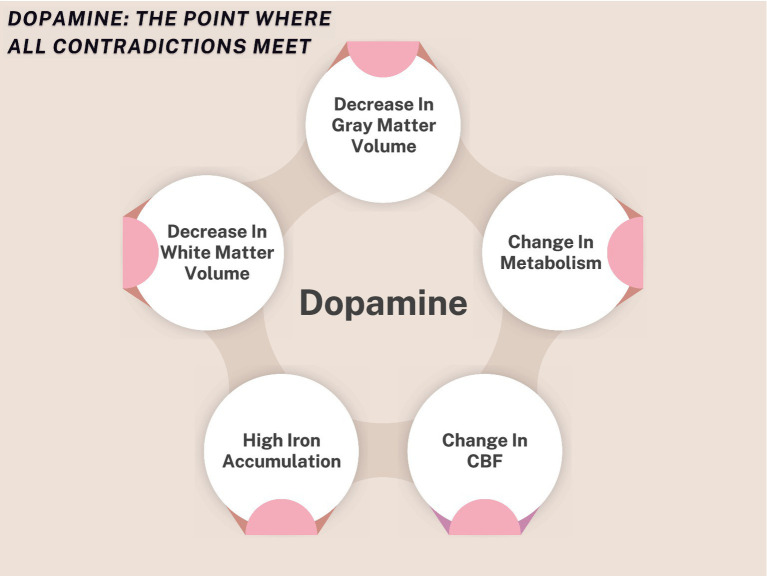
Dopamine as a mechanism accounting for all changes.

Furthermore, dopaminergic signaling can influence white and gray matter development in different ways that may be direct or indirect, depending on developmental stage, regional specificity, and compensatory versus pathological processes (Wood et al., 2009; Hare and Double, 2016; D’Ambrosio et al., 2021). It should be taken into consideration that if dopaminergic dysregulation is proposed as the primary driver of the observed alterations in gray and white matter, such an effect would be difficult to account for unless elevated dopaminergic activity were present early in neurodevelopment. This consideration is particularly relevant given that stuttering typically emerges during early childhood, most commonly between 2 and 5 years of age and in some cases up to 12 years of age. These developmental windows coincide with critical periods characterized by heightened neural plasticity, during which neurotransmitter imbalances can exert disproportionate and long-lasting effects on the maturation, organization, and stabilization of cortical and subcortical circuits. Within this framework, the hypothesis assumes that elevated extracellular dopamine may be present from the onset of the disorder, especially if dopamine is considered an upstream factor capable of shaping the subsequent structural, metabolic, and functional changes observed in PWS (see Figure 2).

Dopamine’s relevance, however, extends beyond its ability to unify physiological changes. It also exhibits an important functional property: the presence of both basal (tonic) and phasic modes of release. Phasic dopamine, in particular, demonstrates extraordinary flexibility. Its magnitude, timing, and target regions fluctuate dynamically in response to emotional states, contextual demands, task requirements, social evaluation, sleep, nutrition, and exposure to various substances (Alm, 2021).

This remarkable variability closely mirrors the situational variability observed in PWS. Because stuttering is inherently unstable, fluctuating across contexts, tasks, emotional states, and time, it is reasonable to infer that its underlying cause is also dynamic rather than fixed. Dopamine, in this regard, appears to be a particularly well-suited component within such a model.

### 7.5 Dopamine as a modulatory mechanism in model 1

In contrast to Alm’s proposal that the dynamics of the dopamine system constitute the main neural basis underlying the situational variability of stuttering, we argue that dopaminergic fluctuations represent one of several interacting contributors to situational variability. Within Model 1 (see Figure 1), dopamine does not act in isolation but instead participates in the process that culminates in the emergence of stuttering.

Specifically, elevated or dysregulated dopamine signaling in the striatum and auditory regions, both of which exhibit abnormalities from early development, directs attention back to the auditory–speech integration pathway. We propose that disrupted dopaminergic signaling within this pathway is a principal driver of aberrant error signals. In this framework, dopamine functions as either a system modulator or destabilizer. When dopaminergic signaling achieves a degree of balance or optimization, the intensity and frequency of error signals are reduced. Conversely, when dopaminergic signaling becomes more dysregulated, error signals intensify, necessitating increased engagement of the SMS and the rIFG.

Thus, dopamine acts as a double-edged mechanism capable of enhancing or degrading system stability (Figure 1). This helps explain why fluency-inducing conditions can lead to dramatic improvements in some individuals, while others continue to stutter to a lesser degree. The determining factor may lie in baseline dopamine levels and, more critically, in the degree of dopaminergic volatility.

Moreover, dopamine provides a plausible explanation for time-based variability, a specific subtype of situational variability. Fluctuations in stuttering severity throughout the day or over longer temporal scales may reflect circadian and state-dependent changes in dopaminergic signaling. Similarly, the shifting pattern of stuttered phonemes and the emergence of new “difficult” words can be interpreted as consequences of ongoing reorganization in dopamine regulation and signaling dynamics.

Our dopaminergic hypothesis may extend beyond formal experimental findings to offer a coherent explanatory framework for recurrent phenomenological observations reported by PWS. Numerous self-reported accounts describe fluctuations in stuttering severity in response to factors such as sleep quality, emotional state, specific foods, alcohol consumption, caffeine intake, and the use of certain supplements or medications. While such observations are inherently subjective and cannot be regarded as direct evidence, consistent directional changes, whether improvement or exacerbation, across these domains may reflect underlying modulation of dopaminergic signaling. Within this framework, noticeable positive or negative shifts in speech fluency in response to these factors are interpreted as indirect manifestations of variability in dopamine release, availability, or receptor sensitivity, thereby contributing to the marked intra-individual and situational variability characteristic of stuttering.

Through the extensive discussion of dopamine dysregulation and its connection to various physiological and clinical variables in stuttering, an important question now arises: What does dopamine dysregulation mean in PWS? What does it signify?

### 7.6 Exploring dopaminergic dysregulation in stuttering: elevated levels and adaptive responses

When discussing *dopamine dysregulation* in stuttering, the critical issue is not merely the absolute level of dopamine, but the brain’s adaptive response to sustained dopaminergic imbalance. Evidence indicates that PWS exhibit markedly elevated extracellular dopamine, approaching a nearly threefold increase relative to neurotypical controls. Such a persistent elevation inevitably necessitates compensatory regulatory mechanisms within the dopaminergic system.

At first glance, this dopaminergic profile appears incompatible with several clinical observations. In fact, many PWS display features that seem inconsistent with dopamine excess, particularly with respect to mood regulation, attention, and other functions typically associated with elevated dopamine signaling. Most prominently, PWS show an increased prevalence of ADHD (Walsh et al., 2025). ADHD is traditionally conceptualized as a disorder of reduced effective dopamine signaling and is most commonly treated with medications that enhance dopaminergic transmission. This apparent contradiction raises a fundamental question:

How can ADHD-like traits emerge in a brain already characterized by elevated extracellular dopamine?

This paradox may be resolved by considering a mechanism well documented in the neurobiology of addiction. In addiction, repeated drug-induced dopamine surges elicit compensatory neuroadaptations. These adaptations include downregulation of postsynaptic dopamine receptors and a progressive reduction in receptor sensitivity, thereby weakening the functional expression of dopaminergic signaling (Volkow et al., 2010, 2016).

In addiction, dopamine levels undergo large, transient surges. Stuttering, by contrast, appears to involve a chronically elevated level of extracellular dopamine, rather than fluctuating peaks and troughs, creating a distinct dopaminergic state.

Chronic elevation of extracellular dopamine in PWS may induce a mild and gradual compensatory reduction in postsynaptic dopamine receptor sensitivity, a process that is likely far less pronounced than the robust receptor downregulation observed in addiction. The result is a paradoxical condition best described as **functional dopamine deficit**: dopamine is present in excess, yet its ability to exert stable and effective signaling is compromised.

In this state, dopaminergic transmission becomes inefficient and unstable. Normal moment-to-moment fluctuations in dopamine release, when acting upon a desensitized receptor system, are more likely to produce inconsistent or distorted signaling. This mismatch—high dopamine availability coupled with reduced functional impact—constitutes what we define as *dopamine dysregulation*, rather than simple hyperdopaminergia or hypodopaminergia.

Importantly, taken together, this model suggests that stuttering represents a unique dopaminergic state: one in which chronic extracellular dopamine elevation paradoxically culminates in functional dopaminergic insufficiency.

Now the most direct and critical question arises: what could be the underlying cause of elevated extracellular dopamine in PWS?

## 8 D2 autoreceptor dysfunction as a candidate mechanism for elevated extracellular dopamine

### 8.1 Introduction to D2 autoreceptors

According to Ford (2014), D2 autoreceptors located on presynaptic dopaminergic neurons in the substantia nigra pars compacta (SNc), VTA, and striatal terminals serve as essential regulators of dopamine signaling through a classic negative feedback mechanism. When activated by extracellular dopamine, these receptors suppress dopaminergic neuron firing, reduce dopamine synthesis by inhibiting tyrosine hydroxylase, and decrease vesicular dopamine release. Ford also highlights that D2 autoreceptors play a role in dopamine clearance by indirectly enhancing dopamine transporter (DAT) function—not through direct binding, but by promoting intracellular pathways that increase DAT surface expression and activity, thereby facilitating more efficient reuptake. This dual action, limiting both release and increasing reuptake, allows D2 autoreceptors to maintain tight control over extracellular dopamine concentrations.

Robinson et al. (2017) further investigated the behavior of D2 autoreceptors after desensitization in midbrain dopamine neurons, particularly focusing on their trafficking and internalization dynamics. Unlike most G protein-coupled receptors (GPCRs), which are typically internalized and recycled or degraded following desensitization, D2 autoreceptors in the substantia nigra were found to resist internalization even after prolonged stimulation. The researchers observed that these receptors remained clustered on the somatodendritic membrane in a punctate distribution pattern. Interestingly, this resistance to internalization was cell type specific. When the same D2 receptors were expressed in non-dopaminergic neurons (e.g., in the locus coeruleus), they internalized normally. This suggests that intrinsic properties of dopamine neurons prevent the removal of desensitized D2 autoreceptors. These observations raise the possibility that, if similar trafficking resistance occurs in pathological states, desensitized D2 autoreceptors could remain at the membrane, potentially altering the dynamics of autoreceptor-mediated feedback control.

### 8.2 Toward a unifying hypothesis: D2 autoreceptor dysfunction in stuttering

We propose that dysfunction in D2 autoreceptors disrupts the brain’s primary mechanism for controlling extracellular dopamine. When these autoreceptors fail to suppress dopamine synthesis, release, and reuptake, extracellular dopamine rises to abnormally high levels. In an attempt to restore balance, the brain downregulates postsynaptic dopamine receptors; however, this adaptive response reduces receptor availability and weakens the efficiency of dopaminergic signaling. Instead of normalizing the system, the combined effect of high extracellular dopamine and diminished receptor responsiveness produces what can be described as a “functional dopamine deficit,” a state in which dopamine is plentiful, yet its signaling impact is unstable, inefficient, or effectively reduced.

Within this framework, several major observations in PWS can be interpreted coherently. Reduced functional dopamine may impair predictive coding and feedforward–feedback matching within the striatum and LSTG, leading to the error-related signals proposed in this framework.

This framework also offers a coherent explanation for the emergence of ADHD-like features in PWS. While the reduction in functional dopamine signaling may not always be severe enough to produce overt symptoms of dopamine deficiency, in certain individuals or developmental contexts it may cross a critical threshold, manifesting clinically as ADHD. This explains why PWS who have high dopamine levels do not exhibit the typical symptoms of high dopamine but rather tend to show conditions associated with inefficient or weak dopaminergic signaling, such as ADHD.

The proposed model may also help explain why CWS are more susceptible to specific difficulties: impaired phonological working memory, attention deficits, challenges in cognitive flexibility, and hyperactive or compulsive behaviors (Anderson and Wagovich, 2010; Alm, 2014; Eichorn et al., 2018; Paphiti and Eggers, 2022). This vulnerability may stem from a functional dopamine deficit, which is likely central to these manifestations given dopamine’s critical role in learning, attention, and compulsive behaviors.

When this theoretical framework is integrated with Figure 1, in which dopamine functions as a system enhancer or destabilizer, our hypothesis and overarching framework are complete. Together, they are proposed to possess the full explanatory capacity to account for situational variability, developmental changes in stuttering symptoms, and the documented physiological alterations associated with the disorder.

The desensitization of presynaptic D2 autoreceptors appears to be the first hidden event that catalyzes everything that follows, as suggested by the hypothesis. However, this process does not represent the root cause of stuttering. These receptors do not lose their sensitivity spontaneously; rather, their desensitization is driven by a specific yet unidentified factor—a mystery element that may hold the key to the true origin of the disorder.

## 9 Empirical tests and falsifiable predictions

### 9.1 Dopamine hypotheses

Regarding the dopamine hypothesis in PWS, the current literature includes only a single study that directly measured dopamine levels. This study, conducted by Wu et al. (1997), examined dopamine activity in only three AWS using PET. Given the extremely small sample size and the lack of replication, the evidence supporting elevated dopamine levels in stuttering remains limited. Therefore, the first and most essential recommendation is to replicate the Wu et al. study in a larger cohort of adults, either using the same PET methodology or alternative techniques with comparable or superior sensitivity and reliability.

This replication is of critical importance, as it constitutes the empirical foundation upon which the dopamine hypothesis is built. Confirming that elevated dopamine levels are consistently present in PWS, and not restricted to a specific subgroup or experimental artifact, would provide strong support for the involvement of dopamine in the pathophysiology of stuttering. Moreover, such studies should aim not only to quantify dopamine levels but also to map the spatial distribution of elevated dopamine within the brain. Identifying the regions where dopamine concentrations are highest would be invaluable for understanding how dopamine may drive the diverse physiological and functional alterations previously discussed across multiple brain regions implicated in stuttering.

Regarding the hypothesis that desensitization or dysfunction of presynaptic D2 autoreceptors contributes to increased extracellular dopamine levels, it is important to note that multiple methods for assessing dopamine function are currently available (Post and Sulzer, 2021; Reneman et al., 2021). Regardless of the specific technique employed, a crucial requirement is that it must be capable of detecting abnormalities specifically related to these autoreceptors. Such abnormalities may include reduced activity, decreased sensitivity, or qualitatively abnormal receptor function. Whatever biomarker or imaging modality is selected, it should provide direct or indirect evidence concerning the functional status of presynaptic D2 autoreceptors in PWS.

One particularly intriguing possibility is that these autoreceptors may be functionally intact at the receptor level, while the underlying dysfunction lies in their regulatory role over dopamine transporter (DAT) expression. Since presynaptic D2 autoreceptors indirectly influence DAT expression, impairment in this signaling pathway could result in reduced DAT availability, thereby leading to elevated extracellular dopamine levels. Consequently, assessing DAT activity or expression in the brains of PWS would be both important and highly informative. However, given their central regulatory role, presynaptic D2 autoreceptors should remain the primary experimental target, particularly within their principal anatomical distribution areas: the ventral tegmental area (VTA), substantia nigra, and striatum.

Measuring dopamine levels in CWS is another area of significant importance. To date, no study in the stuttering literature has directly assessed dopamine levels in pediatric populations. Conducting such research would represent a major advancement in the field. For this to be feasible, any technique used to assess dopamine in children must satisfy two fundamental criteria. First, the method must be safe, as techniques such as PET involve radiation exposure and are therefore unsuitable for use in children. Selecting a non-invasive and safe measurement tool is thus a primary requirement. Second, the method must possess sufficient sensitivity to reliably detect dopamine levels. While extremely high spatial resolution or detailed dopamine mapping is not strictly necessary (although such data would be highly informative), the technique should, at a minimum, be capable of answering a critical question: Is dopamine elevated in the brains of CWS?

Addressing this core question would have profound implications. Demonstrating elevated dopamine levels in CWS would strongly support a causal role for dopamine, indicating that dopaminergic abnormalities are present from the earliest stages of the disorder. Furthermore, if such studies were able to stratify children into subgroups, for example, those with very high dopamine levels, moderately elevated levels, or near-normal levels, and then longitudinally follow these groups into adulthood, it would become possible to test a highly informative hypothesis. Specifically, one could predict that children with relatively lower dopamine elevations would be more likely to recover from stuttering, whereas those with the highest dopamine levels would be more likely to develop persistent stuttering. Such findings would not only deepen our understanding of the neurobiological mechanisms underlying stuttering but could also open new avenues for early prognosis and targeted intervention.

### 9.2 rIFG hypothesis

The proposed hypothesis assigns a primary causal role in the emergence of stuttering to the rIFG. The most compelling evidence in support of this claim would come from real-time measurement of activity across the entire rIFG–HDP–STN specifically during moments of stuttering or immediate speech blocks. Under these conditions, a pronounced and transient spike in rIFG activity is expected, propagating through the HDP to the STN. The detection of such a sharp, time-locked increase in activity would constitute direct evidence for the involvement of this pathway in the generation of stuttering, particularly in blocking phenomena.

During fluent speech, the rIFG–HDP–STN pathway is expected to operate within a normal functional range. Activation during fluent speech may occur at a low level, in a sustained manner, or in a regular rhythmic pattern, reflecting the broader involvement of this pathway in multiple cognitive and motor control processes. However, fluent speech should not be accompanied by sudden, high-amplitude, transient spikes in activity comparable to those observed during stuttering events. Because this pathway cannot be selectively dedicated to speech and necessarily supports a range of functions, baseline or moderate activation during fluent speech is expected and theoretically acceptable. In contrast, abrupt and intense activation should be specific to moments of stuttering. The absence of such stuttering-specific activity would significantly weaken the hypothesis that the rIFG plays a causal role in the emergence of stuttering symptoms.

It remains unclear whether the abnormal connectivity of the rIFG is a consequence of altered extracellular dopamine levels or represents a separate neurobiological abnormality. This uncertainty highlights the importance of investigating the effects of dopamine, particularly during childhood, on the development and connectivity of the rIFG, and of determining whether elevated extracellular dopamine may be a plausible causal contributor to the atypical connectivity patterns observed in this region.

### 9.3 Conscious error monitoring hypothesis

A recent study by Muscarà et al. (2025) introduced a multisensory stimulation protocol that combined delayed and frequency-shifted auditory feedback, visually delayed self-images with color modifications, and vibrotactile input delivered through a specialized vest. These unpredictable stimuli overloaded the SMS and shifted its resources away from internal speech monitoring, leading to immediate fluency improvements and sustained benefits over a 1-year follow-up. By interrupting the overactive monitoring loop, the intervention may have facilitated neuroplastic changes that progressively reduced the involvement of conscious error monitoring in the speech–auditory–motor system, encouraging a shift toward more subconscious speech monitoring. Within our framework, the Muscarà multisensory protocol can be conceptualized as a set of convergent manipulations of the SMS, each transiently reallocating monitoring resources away from speech and, consequently, facilitating fluency. Nevertheless, the underlying neural mechanisms responsible for these improvements remain to be empirically established. Conducting a study based on Muscarà’s approach, which aims to overload the SMS with a high volume of stimuli to prevent the transition from subconscious to conscious error monitoring, would be helpful in confirming the involvement of this system, specifically its conscious component, in the emergence of stuttering. There are many methods that can be used to distract the speaker, and conducting such research or providing creative approaches for this task will not be difficult.

## 10 Future directions

In future work, we will develop and pre-register comprehensive experimental protocols aimed at directly testing the model’s core predictions. These protocols will encompass all major components of the proposed framework, including the hypothesized dysfunction of D2 autoreceptors, the Decode Stuttering Puzzle model (Figure 1), the SMS mechanism, and the involvement of the rIFG. All proposed protocols will be made publicly accessible, enabling independent research groups to reproduce, evaluate, and challenge the hypothesis through standardized and transparent procedures. Such prospective work will provide a rigorous foundation for assessing the model and refining its mechanistic elements.

## 11 Conclusion

In this work, we aimed to present a comprehensive hypothesis capable of explaining the diverse phenomena of stuttering in a logical, coherent, and testable manner. The present study builds upon previous research, hypotheses, and experimental findings, integrating their results into a unified and coherent framework rather than treating them as separate or competing accounts.

In the conclusion, this work revisits dopamine as a primary causal factor or the initiating event from which subsequent pathological processes emerge. Particular emphasis is placed on presynaptic D2 autoreceptors as a potential mechanism underlying the abnormally elevated dopamine levels observed in PWS. In parallel, the study addresses a long-standing debate concerning the role of the rIFG, proposing that this structure may function as both a compensatory and causal mechanism in stuttering, depending on the presence of the warning signals generated by the SMS.

Furthermore, we extend existing models of the SMS by refining and expanding their explanatory scope. We hope that the updated framework is better equipped to account for well-established features of stuttering.

A key contribution of this work is the explicit incorporation of situational variability as a defining feature of stuttering rather than a secondary or peripheral characteristic. By linking situational variability to underlying neurobiological dynamics, we introduce an integrated model (Figure 1) in which dopamine acts as a modulatory factor capable of stabilizing or destabilizing the model across contexts. This framework provides a direct neurobiological explanation for why stuttering severity fluctuates across situations, time, and social environments.

We hope that this work encourages renewed focus on situational variability, dopaminergic mechanisms, the SMS, and the role of the rIFG, and promotes the development of integrative frameworks capable of explaining stuttering as a multidimensional disorder rather than through single-factor accounts.

## Data Availability

No new data were generated or analyzed in this study, as the article is based on theoretical interpretation of previously published literature and proposes conceptual models derived from existing scientific evidence.
